# Bromo-desthiobiotin (Br-DTB) tag for unbiased profiling and identification of chemical modifications

**DOI:** 10.1039/d6cb00192k

**Published:** 2026-10-14

**Authors:** Bibhuti Bhusana Palai, Daniel H. Ramirez, Changfeng Deng, Francisca N. de Luna Vitorino, Chenfeng Zhao, Dong Zhu, Dongwen Lv, Benjamin A. Garcia, Zongtao Lin

**Affiliations:** a Department of Biochemistry and Molecular Biophysics, Washington University School of Medicine in St. Louis MO 63110 USA bagarcia@wustl.edu; b Department of Medicinal Chemistry, Rutgers University Piscataway NJ 08854 USA tom.z.lin@rutgers.edu; c Department of Cellular Biology and Anatomy, Medical College of Georgia, Augusta University Augusta Georgia 30912 USA; d Georgia Cancer Center, Augusta University Augusta Georgia 30912 USA

## Abstract

Leveraging the intrinsic reactivity of cysteine, isoTOP-ABPP (activity-based protein profiling) has been a powerful chemoproteomic platform for cysteine quantification, ligand, and chemical modification discovery. Here, we report new bromo-desthiobiotin azide (Br-DTB) tags for covalent labelling, enabling unbiased identification of chemical modifications. Besides peptide identification from MS2 spectra, Br's isotopic MS1 signatures provide an additional layer of validation to confirm labelled sites and eliminate false-positive assignments. To support this analysis, we have developed a publicly available in-house software to automatically recognize this isotopic signature. The accuracy of the Br-DTB tag was further validated by a synthetic peptide *in vitro* and l-azidohomoalanine (AHA) labelling in living cells. We further elucidated the covalent labelling site of piperlongumine (PL) on the E3 ligase KEAP1, demonstrating its utility for discovering new covalent labelling sites. Collectively, Br-DTB tags are suitable for chemoproteomic workflows, providing a robust platform for covalent labelling discovery and functional proteome profiling.

## Abstract 2

Leveraging the intrinsic reactivity of cysteine, isoTOP-ABPP (activity-based protein profiling) has been a powerful chemoproteomic platform for cysteine quantification, ligand, and chemical modification discovery.

## Graphical abstract

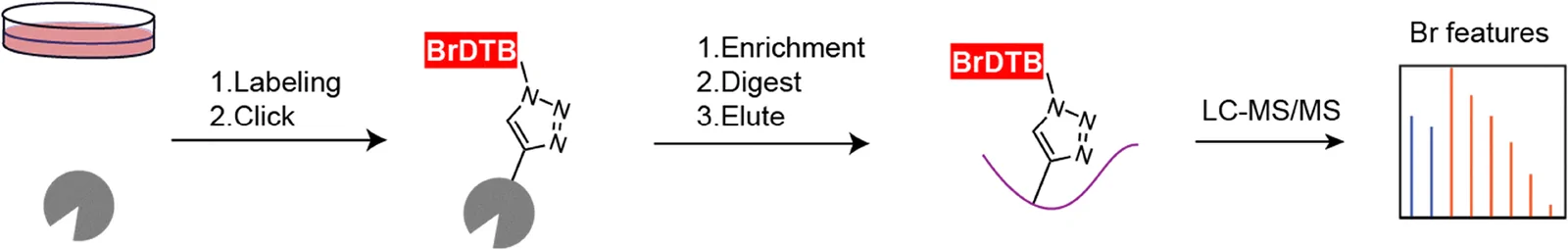

## Introduction

Many studies have sought to elucidate the intrinsic and context-dependent reactivity of amino acid side chains in complex biological environments to guide rational drug discovery.^1,2^ Chemoproteomics enables proteome-wide measurement of amino acid reactivity and ligandability directly in complex biological systems. A widely used strategy for quantifying cysteine reactivity is isotopic tandem orthogonal proteolysis–activity-based protein Profiling (isoTOP-ABPP), which combines cysteine-directed electrophilic probes with isotopically encoded affinity tags to enable quantitative, site-specific analysis by mass spectrometry.^3–5^ The isoTOP-ABPP method has been extended to profile additional residues, including lysine,^6^ methionine,^7^ and tyrosine,^8^ to enable competitive profiling of covalent fragments,^6,9–11^ and mapping ligandable hotspots for fragment-to-lead efforts for “inverse drug discovery.”^12^

Halogenated chemical tags can offer distinct advantages for chemoproteomic analysis because of their natural isotope patterns *e.g.*, bromine (^79^Br/^81^Br ≈ 1 : 1) and chlorine (^35^Cl/^37^Cl ≈ 3 : 1). Several halogen- and isotope-based tagging strategies have been developed for proteomic applications (Fig. 1). Prior work from the Bertozzi lab has combined mono- or di-bromo/chloro isotope tags with computational pattern matching algorithms to detect labeled peptides in complex mixtures.^13^ Building on this concept, dibrominated Isotope-targeted glycoproteomics (IsoTaG) was developed to enable characterization of azide-labeled intact glycopeptides.^14,15^ In addition, isotopically labeled desthiobiotin azide (isoDTB) tags were also used for bacterial cysteinome profiling.^16,17^ More recently, desthiobiotin iodoacetamide (DBAI) based TMT-ABAP workflows have combined desthiobiotin-mediated peptide enrichment with isotopic TMT labeling for quantitative, multiplexed cysteine profiling.^18^ Although these approaches demonstrate the advantage of halogen isotope signatures for selective detection and proteomic analysis, they can involve additional experimental steps. For example, IsoTaG requires chemical cleavage prior to proteomic analysis, whereas isoDTB-based approaches use isotopic tag pairs for quantitative proteomic profiling. These requirements can complicate the workflow when the primary goal is identifying chemical modification sites rather than comparative quantification.

**Fig. 1:**
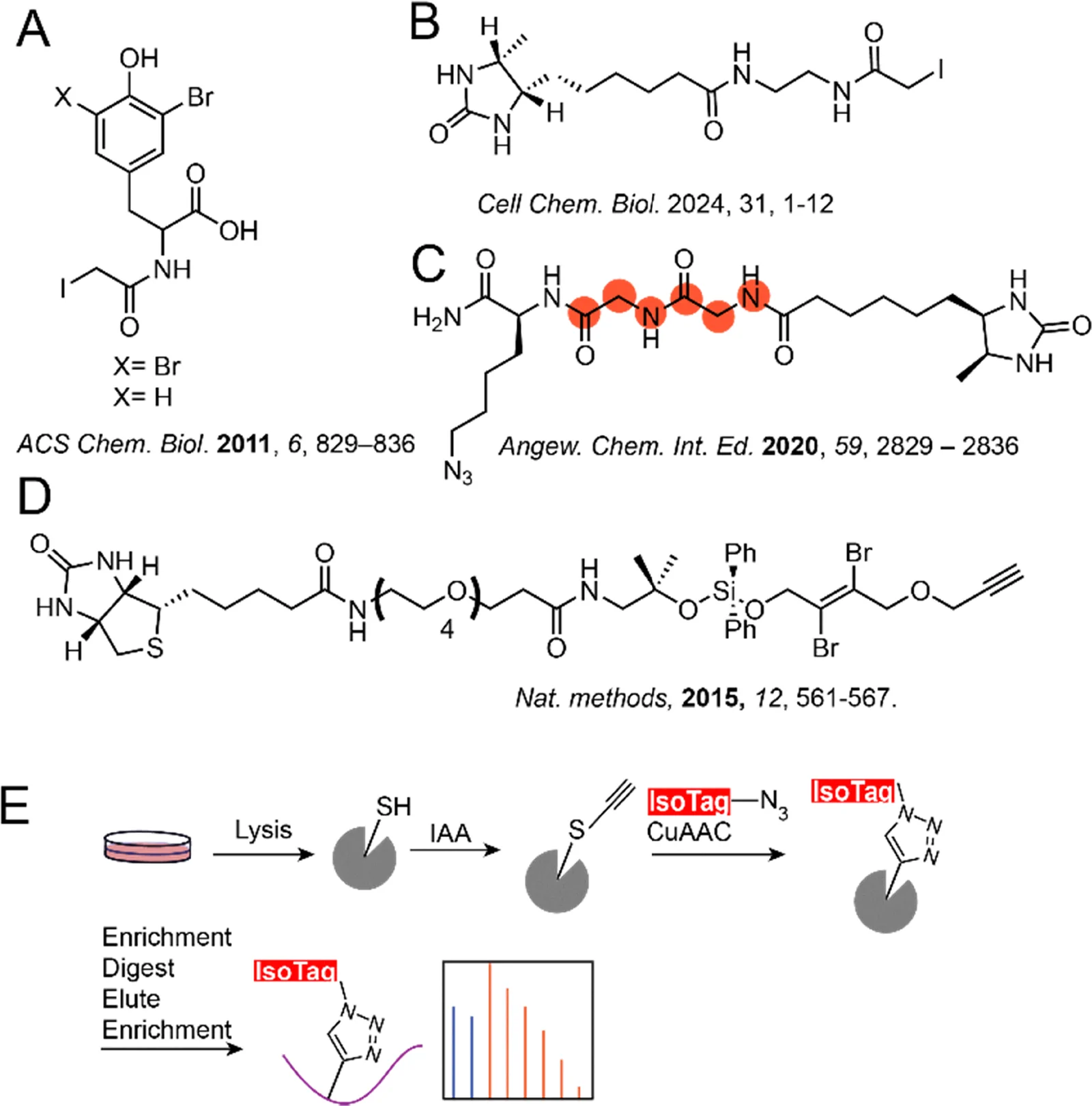
Representative isotopic tags previously used for chemoproteomic profiling: (A) iodoacetamide-derivatized halogen tags from tyrosine, (B) DBIA probe for labeling and enrichment of reactive cysteines, (C) isotopically labeled desthiobiotin (isoDTB) tags for chemoproteomics, highlighted atoms are isotopically labeled, (D) dibrominated silane probe for glycoproteomics, (E) workflow for isoTOP-ABPP.

To address these limitations, we sought to develop a single tag that combines affinity enrichment with an intrinsic isotopic signature for peptide identification. Because the characteristic bromine isotope pattern can be directly detected by mass spectrometry, a pair of tags is not required for identification of chemical modification sites. In addition, using an elutable desthiobiotin moiety instead of biotin can simplify peptide recovery after enrichment. Here, we report the synthesis of a brominated-desthiobiotin tag (Br-DTB) together with a companion pattern-matching software that enables unbiased detection of labeled peptides. The Br-DTB offers a simplified chemoproteomic tag strategy that does not require chemical/enzymatic cleavage, while maintaining isotopic Br signatures for unbiased site identification.

## Experimental

### Cell culture

HEK293T cells, HeLa cells, MCF-7 cells, and HepG2 cells were maintained in DMEM (Gibco, Life), and Jurkat cells (human T lymphocyte cells) were cultured in RPMI 1640 medium (Gibco, Life) supplemented with 10% FBS (Premium South America) and 1% antibiotics (Penicillin–Streptomycin 5000 U mL^−1^) at 37 °C under 5% CO_2_ atmosphere. To harvest, cells were washed twice with 1× PBS, centrifuged, snap-frozen in liquid nitrogen, and stored at −80 °C until lysis.

### In-gel analysis

Frozen HEK293T cell pellets were resuspended in 1x PBS buffer and lysed by sonication. The lysates were centrifuged at 20 000 × *g* for 30 min to remove cell membranes. The concentrations of cell lysate were determined by the BCA protein assay kit using Bovine Serum Albumin (BSA) as the standard and normalized to 1 mg mL^−1^. Cell lysates were separated into four aliquots, each 50 µL, and two additional samples with DMSO as a control. The two lysates was incubated with IAA at 100 µM and 10 µM for 1 hour at 25 °C and then resuspended in 1× PBS buffer to perform the further click reaction (2 mM CuSO_4_, 100 µM Tris[(1-benzyl-1*H*-1,2,3-triazol-4-yl)methyl]amine (TBTA, Sigma-Aldrich), 1 mM Tris(2-2-carboxyethyl)phosphine (TCEP, Sigma-Aldrich), and 100 µM rhodamin-N_3_). Another two lysates were incubated with IAA at 100 µM and 10 µM for 1 hour at 25 °C and then resuspended in PBS buffer to perform the further click reaction (2 mM CuSO_4_, 100 µM Tris(3-hydroxypropyltriazolylmethyl)amine, (THPTA, Sigma-Aldrich), 1 mM Tris(2-2-carboxyethyl)phosphine (TCEP, Sigma-Aldrich), and 100 µM rhodamin-N_3_. After another 1 h, samples were then boiled using 5× SDS-PAGE loading buffer at 95 °C for 5 min and analyzed by 10% SDS-PAGE gels. The samples were diluted into 4× loading buffer, loaded with 10% SDS-PAGE gel, and imaged on a LI-COR ODYSSEY M imager.

### Sample preparation for chemical proteomics

Proteome samples for MS analysis were prepared according to a previously reported method.^19–21^ The HEK293T cells, HeLa, MCF-7, HepG2, and Jurkat cells were washed with 1× PBS buffer and collected by scraping in 1× PBS buffer. The cells were lysed in 1× PBS buffer, centrifuged at 100 000 × *g* for 30 min, and normalized to 1 mg mL^−1^. Each sample (1 mL aliquot) was treated with 10 and 100 µM of probe (IAA). The IAA labeling reaction was incubated at room temperature 1 h. The samples were clicked to the Br-DTB tag by adding 120 µL of a solution consisting of 60 µL of 0.9 mg mL^−1^ TBTA ligand in 4 : 1 *t*BuOH/DMSO, 20 µL of 12.5 mg mL^−1^ CuSO_4_ in water, 20 µL of 14 mg mL^−1^ TCEP in water, and 20 µL of the respective Br-DTB tag in DMSO. The samples are allowed to react at room temperature for 1 h. During the click chemistry reaction, the samples began to precipitate. Once the reactions were completed, 8 mL of cold acetone was added to the sample to precipitate all the proteins. The protein precipitates were centrifuged at 13 000 × *g* at 25 °C for 1 min. The supernatant was removed, and the precipitates were resuspended in 1 mL of cold methanol by sonication. The supernatant was removed after centrifugation at 13 000 × *g* at 0 °C for 1 min. The wash step with methanol was repeated one more time. The pellets were dissolved in 0.5 mL of 8 M urea in 0.1 M triethylammonium bicarbonate (TEAB) buffer to each sample, sonicated to resuspend. Transfer the sample to a 15 mL snap-open tube, add 0.5 mL of PBS to wash the sample tube twice, and transfer PBS to the same 15 mL snap-open tube. 2.5 mL 0.2% NP40/PBS was added to obtain a concentration of 2 M urea. After the 100 µL streptavidin beads were washed with 1× PBS for 3 times, the samples were enriched with beads for 3 h at room temperature.

The beads were centrifuged (1 min, 1000 × *g*) and the supernatant was removed. The beads were resuspended in 600 µL 0.1% NP40 in 1× PBS and transferred to a centrifuge column (Fisher Scientific, 11894131). Beads were washed two times with 0.1% NP40 in 1× PBS, three times with 1× PBS and three times with ddH_2_O. The beads were resuspended in 600 µL 8 M urea in 0.1 M TEAB, transferred to a protein low-binding tube, and centrifuged (1 min, 1000 × *g*). The supernatant was removed, and the beads were resuspended in 600 µL 8 M urea in 0.1 M TEAB. 20 µL of neutralized TCEP (200 mM, 57 mg mL^−1^ neutralize by adding an equal volume of 600 mM K_2_CO_3_ in water) were added and the beads incubated at 37 °C with shaking at 800 rpm for 30 min. Free thiol groups were modified by adding 50 µL of iodoacetamide (74 mg mL^−1^ in water) and incubating at 25 °C with shaking at 800 rpm for 30 min, then centrifuging the beads, and removing most of the supernatant.

For trypsin digestion, 900 µL TEAB were added to the samples after alkylation to obtain a urea concentration of 2 M. Samples were centrifuged (1 min, 1000 × *g*) and the supernatant removed. The beads were resuspended in 200 µL 2 M urea in 0.1 M TEAB. 4 µL 0.5 mg mL^−1^ trypsin (Promega, V5113) were added and the samples were incubated at 37 °C with shaking at 800 rpm overnight. After the digestion, samples were diluted by adding 400 µL of 0.1% NP40 in 1× PBS and transferred to a centrifuge column (Fisher Scientific, 11894131). Beads were washed three times with 0.1% NP40 in 1× PBS, three times with PBS, and three times with water. Peptides were eluted by adding 200 µL 0.1% formic acid in 50% acetonitrile in water to the column. 100 µL of the elution buffer was added twice to the column and peptides were eluted into a Protein LoBind tube by centrifuging at 3000 × *g* for 3 min. The solvent was removed using a vacuum concentrator at 30 °C for approximately 6 h. Samples were dissolved in 20 µL 1% formic acid in water by sonification for 5 min, and transferred into MS sample vials. Samples were stored at −20 °C until LC-MS/MS analysis.

### LC-MS analysis

LC-MS was performed on an Orbitrap Ascend Tribrid Mass Spectrometer (Thermo Fisher Scientific) coupled with a Vanquish Neo UHPLC (for ultrahigh-performance LC) system. Peptide samples were maintained at 7 °C on the sample tray in the LC system. Separation of peptides was carried out on an Easy-Spray PepMap Neo nano-column (2 µm, C18, 75 µm × 150 mm) at room temperature. Mobile phase A was 0.1% Formic acid (FA) in H_2_O, and mobile phase B was 0.1% FA in Acetonitrile. Flow rate was 3 µL min^−1^ and buffer A: 0.1% formic acid in water and B: 0.1% formic acid in acetonitrile. Analysis started with washing in 5% B for 7 min, followed by a gradient from 5% to 40% buffer B in 105 min, an increase to 60% B in 10 min, and another increase to 90% B in 10 min. 90% B was held for 10 min, then decreased to 5% in 0.1 min and held at 5% for another 9.9 min. The injection volume was 5 µL. The voltage applied to the nano-LC electrospray ionization source was 1.9 kV. The temperature of the ion transfer tube (ITC) was set at 275 °C. Spectra were collected in a data-dependent acquisition mode with MS1 scan range of *m*/*z* 350–2000 in the Orbitrap.

### Peptide identification and quantification

The MS/MS spectra were extracted from the raw file generated by the instrument (Xcalibur software) and searched using Byonic software v.4.5.2 (Protein Metrics) against a reverse-concatenated, non-redundant database of the human proteome (20 398 proteins in total, from UniProt). The mass tolerances of precursor and product ions were set to 10 ppm and 20 ppm, respectively. Cysteine carbamidomethylation (+57.0214 Da) was chosen as a static modification, and oxidation (+15.99492 Da) on methionine residues was allowed for two per peptide as a ‘common’ variable modification. 658.2509 Da (C_30_H_43_BrN_8_O_4_) Br-DTB tag is set as a variable modification on cysteine.

### Synthetic peptide validation of Br-DTB labelling

A synthetic 14-mer peptide (LVVPATQCGSLIGK) containing a single cysteine residue was purchased from GenScript, resuspended in ultrapure water, and stored at −20 °C until use. The peptide (10 µg) was incubated with iodoacetamide-alkyne (IAA, 10 µM) for 1 h at room temperature to selectively alkylate the cysteine thiol. Following alkylation, the alkyne-labeled samples were conjugated to the Br-DTB tag *via* Cu(i)-catalyzed azide–alkyne cycloaddition (CuAAC). For the click reaction, 12 µL of a freshly prepared solution was added, consisting of 2 µL of 0.9 mg mL^−1^ TBTA ligand in 4 : 1 *t*BuOH/DMSO, 2 µL of 12.5 mg mL^−1^ CuSO_4_ in water, 2 µL of 14 mg mL^−1^ TCEP in water, and 2 µL of 5 mM Br-DTB tag in DMSO. The reaction mixtures were allowed to proceed at room temperature for 1 h. Aliquots collected before and after the CuAAC reaction were analyzed by LC–MS. The resulting mass spectra showed the expected mass shift corresponding to cysteine alkylation by IAA and a further characteristic increase in mass following Br-DTB conjugation, confirming efficient and specific labeling of the peptide.

### Metabolic labeling by l-AHA

HEK293T cells were cultured in DMEM supplemented with 10% fetal bovine serum (FBS) and grown to approximately 70–80% confluency. For metabolic labeling, cells were washed once with warm 1× PBS and incubated in methionine-free DMEM containing 10% dialyzed FBS for 30–60 min at 37 °C to deplete intracellular methionine. Cells were then labeled with l-azidohomoalanine (AHA; 50 µM final concentration) in methionine-free DMEM for 2–4 h at 37 °C. Labeling was terminated by washing cells twice with cold PBS, followed by lysis in SDS buffer. Protein concentrations were determined prior to click chemistry. AHA-labeled proteins were conjugated to Br-DTB-alkyne probes *via* copper(i)-catalyzed azide–alkyne cycloaddition (CuAAC). Labeled proteins were subsequently enriched using streptavidin beads, then denatured, reduced, alkylated, and digested with trypsin. The resulting peptides were collected for mass spectrometry (MS)-based proteomic analysis, enabling identification of AHA-labeled peptides modified with the Br-DTB probe.

### PL-probe labeling of Keap1

HEK293T cells were plated at a cell density of 3 × 10^6^ cells per plate in a 10 cm plate and left in a humidified incubator 5% CO_2_ overnight. The HEK293T cells were transfected at 70% confluency with a Keap1 plasmid containing a C-terminal Flag tag using PEI (30 µg/10 cm dish), plasmid (10 µg/10 cm dish), Opti MEM (1 mL), and left to incubate for 48 hrs. Keap1 transfected cells were treated with 20 µM final concentration of piperlongumine (PL) alkyne for four hours. Cells were collected by scraping, pelleted, and lysed in 500 µL of 1X RIPA lysis buffer containing 1X protease inhibitors. Keap1 was enriched using anti-Flag (M2) agarose beads (Sigma), and then Br-DTB-N_3_ was clicked to the enriched labeled Keap1 as described above. The beads were washed to remove excess Br-DTB-N_3_ compound and Keap1 was reduced before trypsin digestion. Peptides were collected and analyzed by mass spec as described above.

## Results and discussion

Brominated desthiobiotin (Br-DTB) tag (Fig. 2A) was synthesized in three steps from commercially available 4-bromophenyl-Boc amino propanoic acid with the synthesis route (Fig. S1–S4). Using analogous conditions, azide- (Br-DTB-Azide) and alkyne-functionalized (Br-DTB-Alkyne) probes were also synthesized for different purposes (Fig. S5–S8).

**Fig. 2:**
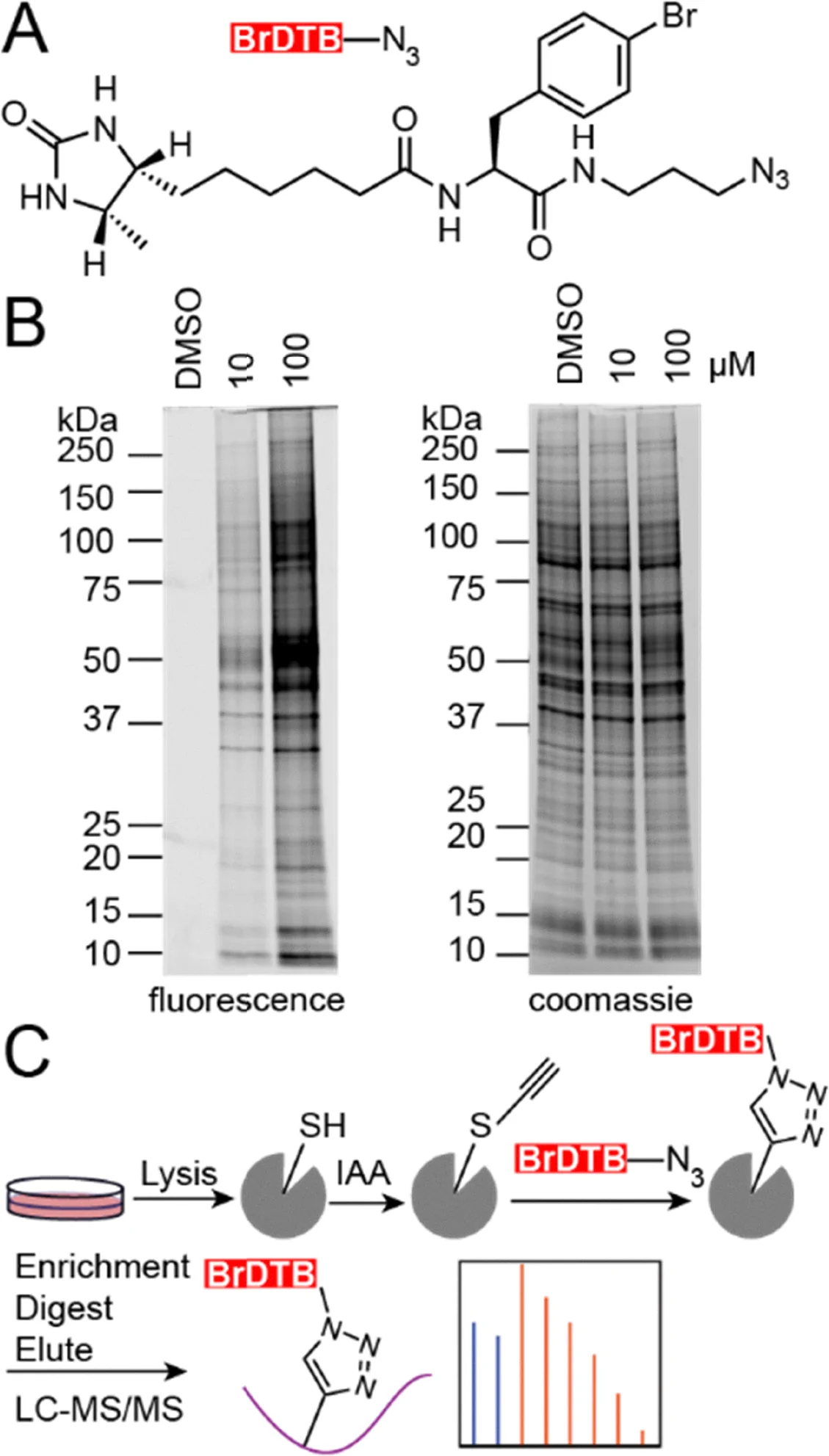
(A) Structure of bromo-desthiobiotin (Br-DTB) probe. (B) IA-Alkyne labeling of the HEK293T Proteome by the gel-based ABPP. (C) Workflow for isoTOP-ABAP using the Br-DTB tag.

To evaluate IAA labeling efficiency, we assessed cysteine labeling in HEK293T cell lysates by in-gel fluorescence. Soluble fractions of cell lysates (1.0 mg mL^−1^) were treated with 10 µM or 100 µM iodoacetamide alkyne (IAA), followed by conjugation to a fluorescent rhodamine–azide tag and analysis by SDS–PAGE. In-gel fluorescence imaging (Fig. 2B) revealed a markedly stronger global labeling signal at 100 µM relative to 10 µM, consistent with enhanced cysteine coverage at higher probe concentrations.

We next applied our chemical tag to profile cysteine modifications after IAA labeling in HEK293T cells (Fig. 2C). Across datasets (Fig. 3A and B), Br-DTB–labeled peptides were robustly identified and profiled under both dosing conditions. Labeling was near-complete at 100 µM IAA, whereas 10 µM IAA selectively captured a subset of cysteine labeling sites. In the soluble fraction, we identified 2125 Br-DTB–labeled cysteine sites at 100 µM IAA and 464 sites at 10 µM IAA. In the membrane fraction, 1132 sites were detected at 100 µM and 323 sites at 10 µM. As expected, higher IAA concentrations resulted in broader cysteine coverage. Applying the same workflow to additional four human cell lines yielded comparable proteomic depth.

**Fig. 3:**
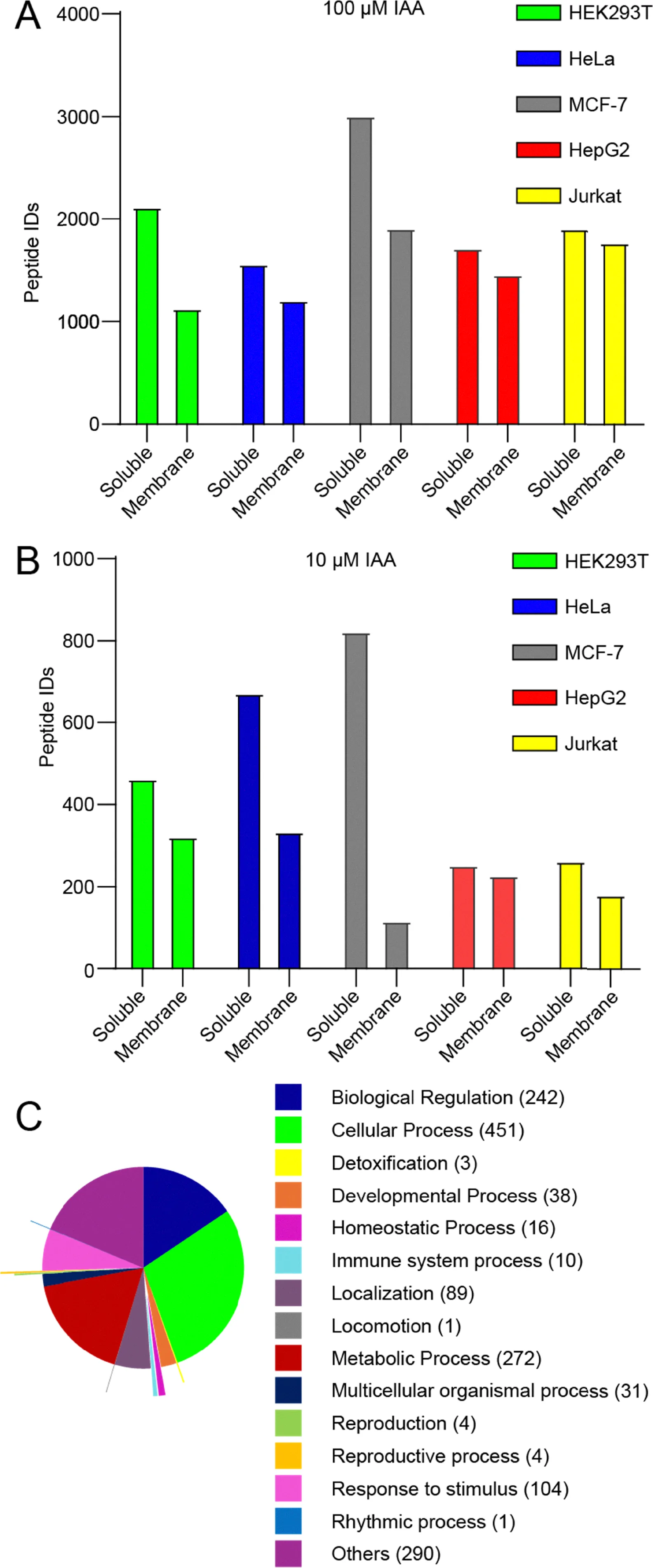
(A) and (B) Comparison of the number of cysteines labeled with Br-DTB peptide under low (10 µM) and high (100 µM) concentrations, (C) biological function analysis of Br-DTB protein substrates using PANTHER.

We analyzed a representative dataset derived from the soluble fraction of HeLa cells treated with 100 µM IAA using PANTHER. Among the 1566 proteins identified, 897 protein targets were assigned to major biological processes. The largest category comprised proteins involved in core cellular processes (451 proteins). There are also significant contributors associated with biological regulation (242 proteins). A substantial number of proteins were also mapped to cellular and metabolic pathways (272 proteins), reflecting the broad metabolic activity captured by our profiling approach. Collectively, these results demonstrate that the Br-DTB peptide profiling strategy robustly identifies IAA labeling sites in proteins spanning essential biological functions (Fig. 3C).

To illustrate the distinctive features of Br-DTB–modified cysteine-containing peptides, we present a representative example from the labeling sites of HEK293T cells: LVVPATQCC[+658.259]GSLIGK (*m*/*z* = 2044.03). The MS1 spectrum clearly displays the characteristic bromine isotopic pattern of the Br-DTB–labeled peptide (Fig. S9A). Two precursors at different charge states corresponding to this peptide were observed: *m*/*z* 682.66 (*z* = 3) and *m*/*z* 1023.52 (*z* = 2). MS/MS fragmentation confirmed the presence and localization of the Br-DTB modification (Fig. S9B).

To orthogonally validate Br-DTB–based profiling, we purchased a corresponding synthetic peptide (LVVPATQCGSLIGK) (Fig. S10 upper), which was then labeled by IAA (Fig. S10 middle), and CuAAC conjugation with the Br-DTB–azide (Fig. S10 bottom). The resulting spectra resolved three distinct species: the unmodified peptide, the IAA-modified peptide, and the Br-DTB–conjugated product, with the latter exhibiting the characteristic ^79^Br/^81^Br isotopic envelope. These well-resolved MS1 signatures (Fig. S10) confirmed the labeling mechanism by IAA and Br-DTB. MS2 spectra are provided in Fig. S11.

To reduce false-positive identifications, diagnostic MS1 bromine isotope patterns were used to validate chemical modifications on peptides. An in-house computational tool (publicly available on GitHub) was developed to automatically recognize isotope signatures and filter candidate peptides. The software, BrMatcher, is Python-based, and it iteratively computes and compares similarity scores between experimental MS1 spectra and theoretical isotope distributions corresponding to bromo- and non-bromo–labeled peptide compositions. The overall workflow is illustrated in Fig. 4A. Briefly, the software parses an Excel output file generated by the mass spectrometer, which contains the peptide sequence, charge state, data file name, and retention time. Each unique combination of peptide sequence, charge state, and data file defines a peptide object. The pipeline iterates over all peptide objects, skipping those already processed, and for each candidate extracts the corresponding experimental MS1 spectrum from the mzXML file. Similarity scores are then calculated against computed isotope patterns for both Br-DTB–modified and unmodified peptide species, enabling confident discrimination of brominated peptides. A step-by-step operation manual of the software is provided as SI. An important feature of this software is that it freely allows users to add customized modifications of interest and associated chemical formulas.

**Fig. 4:**
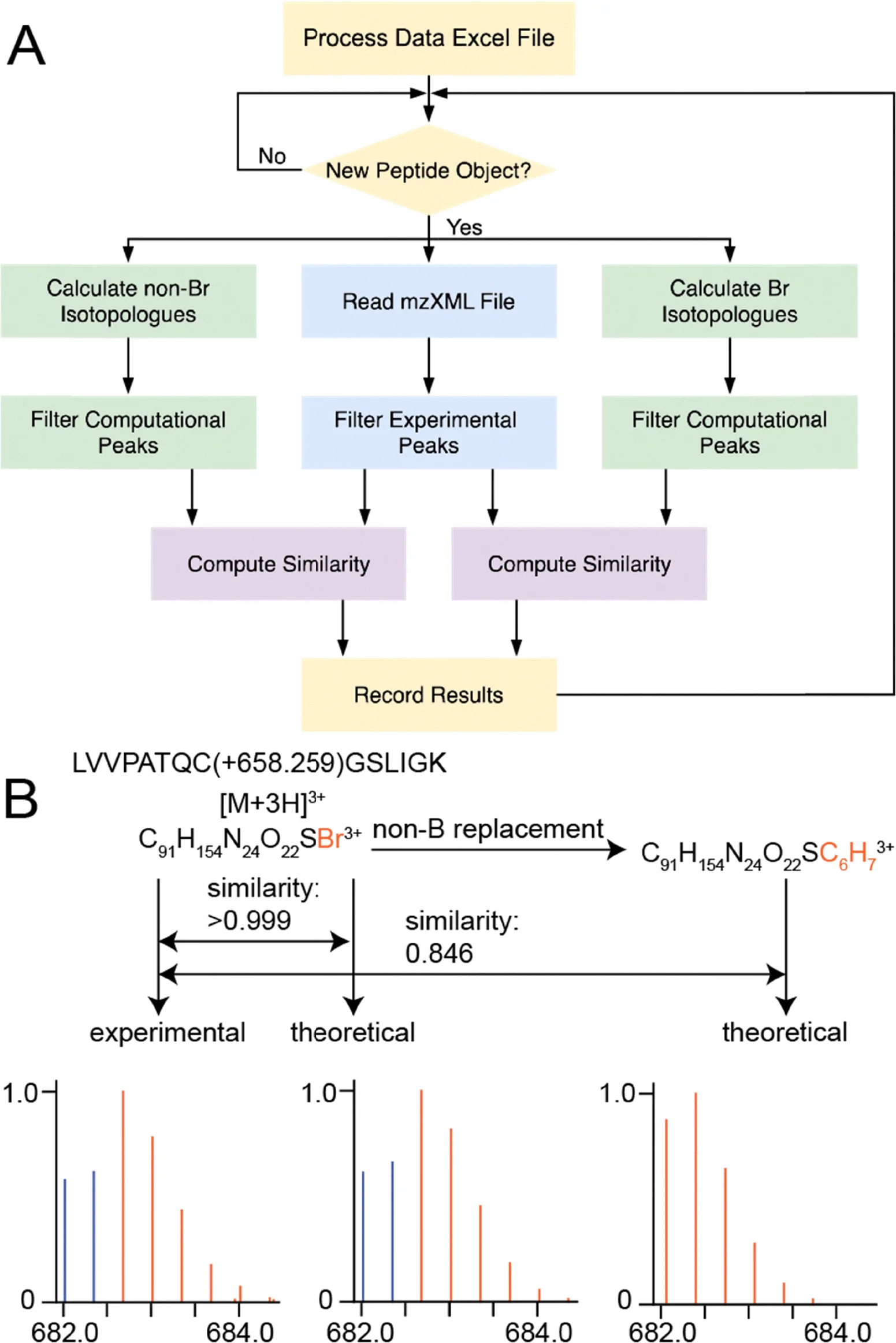
(A) Workflow diagram of the software BrMatcher for chemical modifications containing Bromo tag. (B) Software analysis of an example peptide LVVPATQC[+658.259]GSLIGK. Left: Normalized experimental spectrum. Middle: Normalized theoretical spectrum (Br), similarity: >0.999. Right: Normalized theoretical spectrum (non-Br), similarity: 0.846.

For the generation of computational spectra (Fig. 4B), isotopologue peak distributions for both Br-labeled and non–Br-labeled peptide compositions were first calculated using the proteomics library based on the specified charge state. Using LVVPATQC(+658.259)GSLIGK as an example, its theoretical MS1 spectrum with and without a Br atom is displayed in the middle and right panels of Fig. 4B. For experimental spectra (Fig. 4B), the software reads the corresponding mzXML file (as specified by the data file name) and extracts all MS1 scans with retention times within ±0.5 min of the value reported in the Excel file. Each qualifying scan is treated as an independent experimental spectrum. The left panel of Fig. 4B shows a representative experimental MS1 scan. Similarity scores between each experimental spectrum and the two corresponding computational spectra (Br-labeled and non–Br-labeled) are then calculated using the cosine greedy algorithm implemented in the MatchMS library. The resulting scores are compared to determine which theoretical spectrum matches closer with the experimental data, thereby indicating the likely presence or absence of the brominated (Br-DTB) tag. The example peptide has a similarity of >0.999 when compared with a theoretical MS1 containing a Br, bigger than a similarity of 0.846 when compared with non-Br theoretical MS1 spectrum. Detailed spectra are provided in Fig. S12.

To complement cysteine reactivity profiling, we also mapped newly synthesized proteins incorporating l-azidohomoalanine (AHA), a methionine analog bearing an azide handle that enables bioorthogonal noncanonical amino acid tagging (BONCAT).^22^ To support this application, we synthesized the Br-DTB–alkyne tag using a workflow shown in Fig. 5A. HeLa cells were pulsed with AHA to incorporate the azide at methionine positions during translation. After data analysis, 186 Br-DTB-labeled peptides were identified in the soluble fraction, and 27 Br-DTB peptides were detected in the membrane fraction. A representative peptide SGSM[+485.172]DPSGAHPSVR was used to illustrate the characteristics of the bromine signature of AHA-labeled Br-DTB peptide (Fig. 5A). The precursor ion was observed at *m*/*z* 623.93, with a charge state *z* = 3. Its MS1 (including similarity check) and MS2 spectra are shown in Fig. S13.

**Fig. 5:**
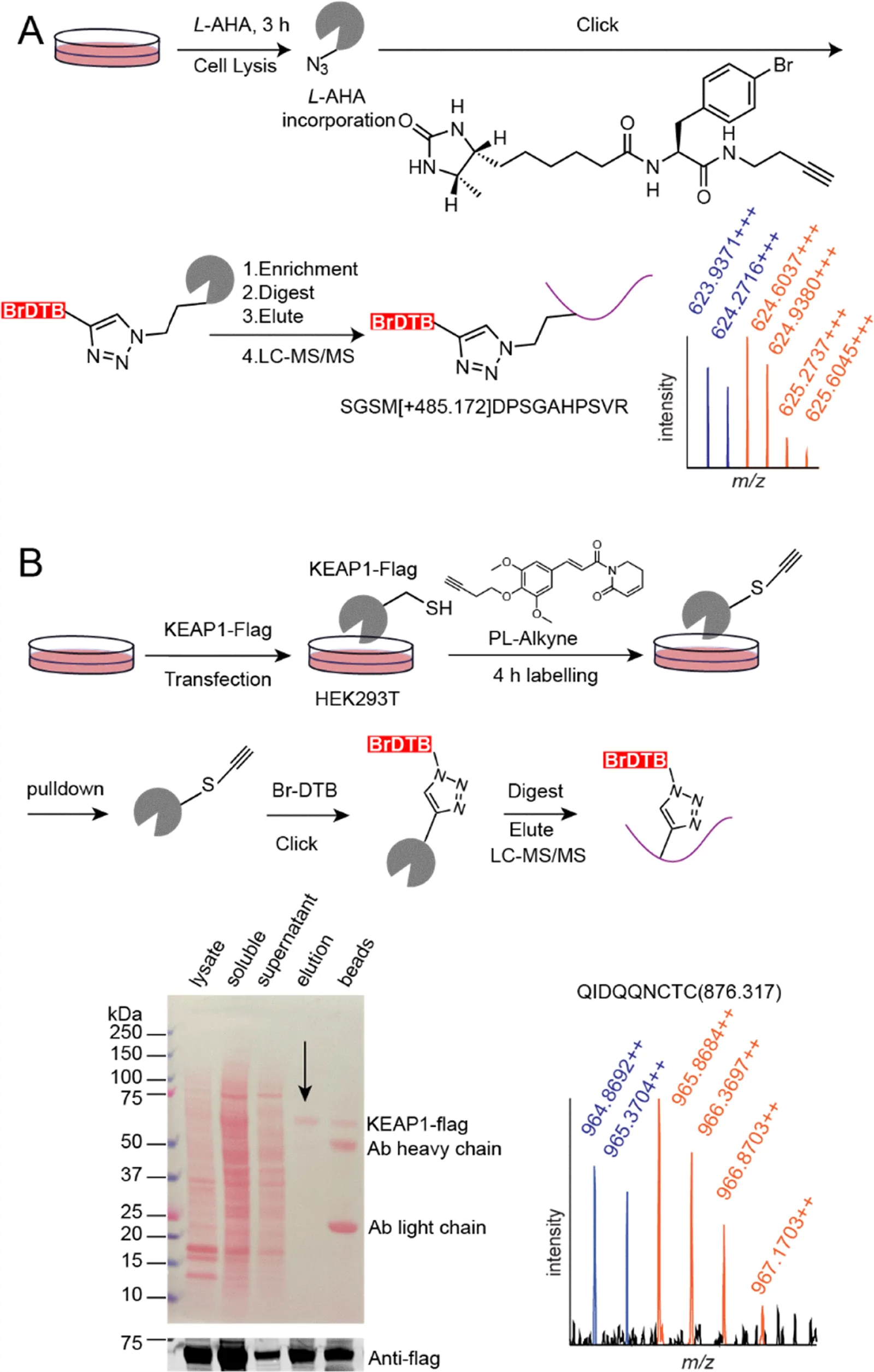
Chemical modification using Br-DTB tags. (A) Workflow of l-AHA incorporated into a *de novo* synthesized protein and tagged with Br-DTB-Alkyne by click chemistry. Representative mass spectra of l-AHA incorporated Br-DTB-Peptide SGSM[+485.172]DPSGAHPSVR. (B) Experimental workflow for PL binding-site elucidation enabled by the Br-DTB probe. Chemical structure of Piperlongumine-Alkyne (PL-Alkyne), immunoprecipitation of KEAP1 and western blot, and MS1 spectrum of PL-alkyne modified Br-DTB peptide QIDQQNCTC[+876.317].

Another application of the Br-DTB tag is to discover unknown covalent chemical modifications between small molecules and proteins. Kelch-like ECH-associated protein 1 (KEAP1) functions as a substrate adaptor for the CUL3–RBX1 E3 ubiquitin ligase complex, where it plays a central role in substrate recognition and ubiquitination.^23,24^ Piperlongumine (PL) is a natural product bearing α,β-unsaturated carbonyl groups and acts as an electrophile capable of covalently modifying nucleophilic cysteines in KEAP1. However, the specific binding site of PL on KEAP1 had not been elucidated. To address this question, we first overexpressed KEAP1 in cells (Fig. 5B), then treated cells with PL probe for 4 hours. The PL-modified KEAP1 was then pulled down by affinity purification (Fig. 5B), going through a click reaction with a complementary Br-DTB–azide tag. Proteins were denatured, reduced, and digested with trypsin. The resulting peptides were analyzed by LCMS-based proteomics (*n* = 2). This analysis revealed a Br-DTB–modified peptide, QIDQQNCTC[+876.317], with a precursor ion observed at *m*/*z* 694.86 (*z* = 2) (Fig. 5B). Detailed MS2 spectra and similarity check by BrMatcher are provided in Fig. S14. Importantly, this result localizes PL modification to a cysteine residue at the C-terminal region of KEAP1 (Fig. S15).

## Conclusion

A new brominated desthiobiotin azide/alkyne (Br-DTB) tag was designed, synthesized, and demonstrated its broad utility in chemoproteomic applications. The Br-DTB tagging systematically identified cysteine reactivity across the human proteome in multiple cell lines, and l-azidohomoalanine (AHA)–based BONCAT labeling enabled selective enrichment of nascent proteomes *via* click chemistry. Br-DTB enabled discovery of piperlongumine (PL)-mediated covalent labeling of KEAP1, demonstrating its capability in covalent chemistry discovery. In addition, we developed a Python-based computational tool that automates the identification of brominated peptides by iteratively comparing experimental and theoretical isotope patterns, thereby improving confidence and reducing false-positive assignments. Together, Br-DTB tag is an easy-to-use and cost-effective platform for unbiased identification of chemical modifications.

## Author contributions

BP performed proteomics experiments and data analysis, DR performed KEAP1 experiments, BP and CD drafted the manuscript, FV did cell culturing, CZ wrote software, DZ and DL provided materials for KEAP1 experiments. BG and ZL conceived the project. All authors reviewed and approved the manuscript.

## Conflicts of interest

There are no conflicts to declare.

## Supplementary Material

CB-OLF-D6CB00192K-s001

## Data Availability

Supplementary information (SI) is available online. Raw data are available on Massive. (Username: MSV000101416_reviewer, Password: b59282aZ43Yg260t). Br-Matcher software code is available on GitHub: https://github.com/ChenfengZhao/BrDTB. See DOI: https://doi.org/10.1039/d6cb00192k.
