# Supplementary material for: Bromo-desthiobiotin (Br-DTB) tag for unbiased profiling and identification of chemical modifications
Source: RSC Chem Biol. 2026 Oct 14. Online ahead of print. doi: 10.1039/d6cb00192k (PMC13682531; doi:10.1039/d6cb00192k)
Supplement: Supplementary file 1 [file CB-OLF-D6CB00192K-s001.pdf]

Supplementary Information (SI) for RSC Chemical Biology.
This journal is © The Royal Society of Chemistry

Supporting information

Bromo-Desthiobiotin (BrDTB) Tag for Unbiased Profiling and Identification of Chemical Modifications

Bibhuti Bhusana Palai[a], Daniel H. Ramirez[a], Changfeng Deng[b], Francisca N. de Luna Vitorino[a], Chenfeng Zhao[b], Dong Zhu[c,d], Dongwen Lv[c,d], Benjamin A. Garcia[a],*, Zongtao Lin[b],*

[a] Department of Biochemistry and Molecular Biophysics, Washington University School of Medicine in St. Louis, MO 63110, USA

[b] Department of Medicinal Chemistry, Rutgers University, Piscataway, NJ 08854, USA.

[c] Department of Cellular Biology and Anatomy, Medical College of Georgia, Augusta University, Augusta, Georgia, 30912, USA

[d] Georgia Cancer Center, Augusta University, Augusta, Georgia, 30912, USA

*Correspondence: ZL (tom.z.lin@rutgers.edu), BAG (bagarcia@wustl.edu)

Table of Contents

1. General Chemistry Procedures....4

2. Experimental Procedures and Analytical Data....4

3. NMR($^1H/^{13}C$) of compound (2)....7

4. NMR($^1H/^{13}C$) of compound (3)....8

5. NMR($^1H/^{13}C$) of compound (4)....10

6. ESI/MS Spectra of compound (4)....11

7. NMR($^1H/^{13}C$) of compound (2a)....12

8. NMR($^1H/^{13}C$) of compound (3a)....13

9. NMR($^1H/^{13}C$) of compound (3a)....15

10. ESI/MS Spectra of compound (4a)....16

11. Synthetic peptide labeled BrDTB and its MS1 and MS2 spectra....19

12. Establishment of in-house Br-Matcher software for Br-Isotopic distribution peptide analysis....20

13. Example of an AHA-labeled BrDTB peptide and its MS1 and MS2 spectra....21

14. Representative MS1 and MS2 spectra of PL-alkyne modified BrDTB peptide....24

15. The KEAP1–CUL3–RBX1 dimer complex structure binds to the C-terminus of KEAP1....26

16. Instructions for running Br-Matcher for Windows OS....27

17. RAW file information....28

18. Raw gels/blots....29

19. Pulse-and-Chase Experiment: Br-DTB Labeling Followed by Rhodamine-Azide Click Reaction....32

20. Biological Replicates of Br-DTB Chemoproteomic Profiling....33

1. General Chemistry Procedures

All the chemical reactions sensitive to air and moisture were carried out in oven-dried glassware under an argon atmosphere using standard Schlenk techniques. Commercially available chemicals were used without further purification unless otherwise mentioned. All reactions were monitored by thin-layer chromatography (TLC), which was conducted on Merck silica gel plates (0.25 mm thick, 60F254), visualized by using UV (254 nm). Silica gel chromatography was performed on a Teledyne ISCO CombiFlash purification system using prepacked silica gel columns (12–330 g sizes). ^{1}H NMR spectra were obtained on a Varian 400 MHz NMR instrument equipped with an autosampler. The chemical shifts (δ) are reported as δ ppm relative to TMS using the residual solvent peak as the reference unless otherwise noted. The following abbreviations are used to express the multiplicities: s = singlet; d = doublet; dd = doublet of doublets; t = triplet; q = quartet; m = multiplet; br = broad. High-performance liquid chromatography (HPLC) was carried out on a GILSON GX-281 using a Waters C18 5 µM, 4.6 × 50 mm and Waters Prep C18 5 µm, 19 × 150 mm reverse phase column, eluting with a gradient system from 5:95 to 95:5 acetonitrile: water with a buffer consisting of 0.05% TFA. Mass spectra (MS) were generated on an Agilent or Waters HPLC/MSD using electrospray ionization (ESI) for detection.

2. Experimental Procedures and Analytical Data

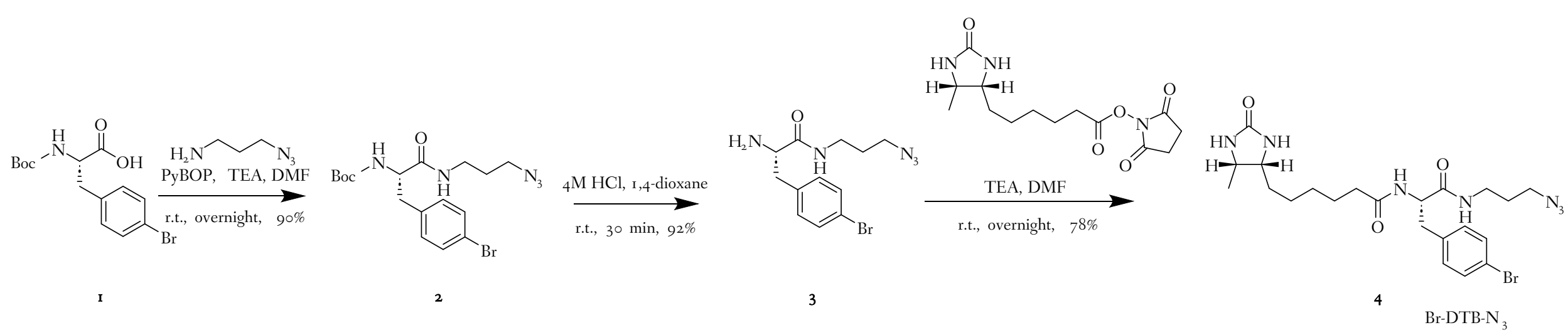

Step 1. tert-butyl (S)-(1-((3-azidopropyl)amino)-3-(4-bromophenyl)-1-oxopropan-2-yl)carbamate (2)**:** To a stirred solution of acid **1** (500 mg, 1.45 mmol) in anhydrous DMF (4 mL) was added PyBOP (760 mg, 1.45 mmol, 1.0 equiv.), azidopropylamine (134 ul, 1.45 mmol, 1.0 equiv.) and DIPEA (19 ul, 1.45 mmol, 1.0 equiv.) at 0 °C. The reaction mixture was allowed to warm up to r.t. and stirred overnight for ~12 h. After completion of the reaction was monitored by TLC and the Solvent was removed under reduced pressure, and the mixture was added saturated Na_2CO_3 (20 mL) in H2O and stirred for 5 min. EtOAc was used for extraction (3 × 20 mL), the combined solvent was washed with brine (20 mL) and H_2O (20 mL) and dried over Na_2SO_4. The combined organic solution was subjected to flash chromatography under gradient conditions (0-40% EtOAc in hexane) to afford amide (560 mg, 1.37 mmol, 90%) as a white solid. ^{1}H NMR (400 MHz, $CDCl_3$) δ 7.43 (d, J = 8.0 Hz, 2H), 7.08 (d, J = 8.0 Hz, 2H), 6.16 (s, 1H), 5.06 (s, 1H), 4.26 (d, J = 7.8 Hz, 1H), 3.26 (h, J = 6.5 Hz, 4H), 3.00 (d, J = 7.0 Hz, 2H), 1.68 (p, J = 7.4 Hz, 2H), 1.41 (s, 9H), ^{13}C NMR (100 MHz, $CDCl_3$) δ 174.55, 171.06, 135.69, 131.73, 131.05, 55.79, 49.01, 37.84, 36.97, 28.51, 28.24.. MS (ESI$^+$) *m/z* calc'd for $C_{17}H_{24}BrN_5O_3$ $[M+H]^+$: 426.1, found 426.5.

Step 2. (S)-2-amino-N-(3-azidopropyl)-3-(4-bromophenyl)propenamide (3)**:** Hydrochloric acid (4 M HCl in H_2O:1,4-dioxane (1:3, 4 mL)) was added dropwise to **2** (480 mg, 1.12 mmol) at 0 °C. The resulting mixture was stirred for ~2 h at room temperature. After completion of the reaction, the reaction mixture Solvent was removed

under reduced pressure, and the mixture was washed with diethyl ether (3 × 5 mL) and dried to afford amine **3** as an HCl salt as white solid (375 mg, 1.04 mmol, 92%). The crude product was used for the next step without further purification. ^{1}H NMR (400 MHz, CD_3OD) δ 7.56 – 7.47 (m, 2H), 7.26 – 7.17 (m, 2H), 4.03 (t, J = 7.2 Hz, 1H), 3.43 – 3.00 (m, 6H), 2.32 (s, 2H), ^{13}C NMR (100 MHz, CD_3OD) δ 168.01, 133.43, 131.78, 131.09, 121.37, 80.50, 69.75, 54.03, 38.25, 36.64, 18.19. MS (ESI$^+$) *m/z* calc'd for $C_{12}H_{16}BrN_5O$ $[M+H]^+$: 326.1, found 326.5.

Step 3. N-((S)-1-((3-azidopropyl)amino)-3-(4-bromophenyl)-1-oxopropan-2-yl)-6-((4R,5S)-5-methyl-2-oxoimidazolidin-4-yl)hexanamide (4)**:** To a stirred solution of salt **3** (375 mg, 1.04 mmol) in anhydrous DMF (10 mL) was added TEA (283 μL, 20.08 mmol, 2.0 equiv.) and desthiobiotin NHS ester (323 mg, 0.21 mmol, 1.0 equiv.) at 0 °C. The reaction mixture was allowed to warm up to r.t. and stirred overnight for ~12 h. Solvent was removed under reduced pressure, and the mixture was subjected to flash chromatography under gradient conditions (0-5% MeOH in DCM). Fractions containing the product were combined, dried, and subjected to flash chromatography (C18 column) under gradient conditions (20-100% MeOH in H_2O) to afford product (424 mg, 0.81 mmol, 78%) as a white solid. ^{1}H NMR (400 MHz, $CDCl_3$): δ 7.96 (d, J = 8.2 Hz, 1H), 7.35 (d, J = 7.9 Hz, 2H), 7.24 (s, 1H), 7.08 (d, J = 8.0 Hz, 2H), 4.74 (q, J = 7.9 Hz, 1H), 3.90 (t, J = 6.9 Hz, 1H), 3.76 (d, J = 9.7 Hz, 1H), 3.36 – 3.13 (m, 4H), 2.96 (qd, J = 13.7, 7.4 Hz, 2H), 2.18 (qt, J = 13.9, 6.9 Hz, 2H), 1.78 – 0.97 (m, 13H), ^{13}C NMR (100 MHz, $CDCl_3$) δ 174.55, 173.79, 172.09, 136.01, 131.47, 131.05, 120.60, 56.47, 54.34, 51.80, 48.80, 37.43, 36.70, 34.95, 29.56, 28.49, 27.81, 25.78, 25.11, 15.80. Purity: 99.1% determined by UPLC. MS (ESI$^+$) *m/z* calc'd for $C_{22}H_{32}BrN_7O_3$ $[M+H]^+$: 522.2, found 522.5.

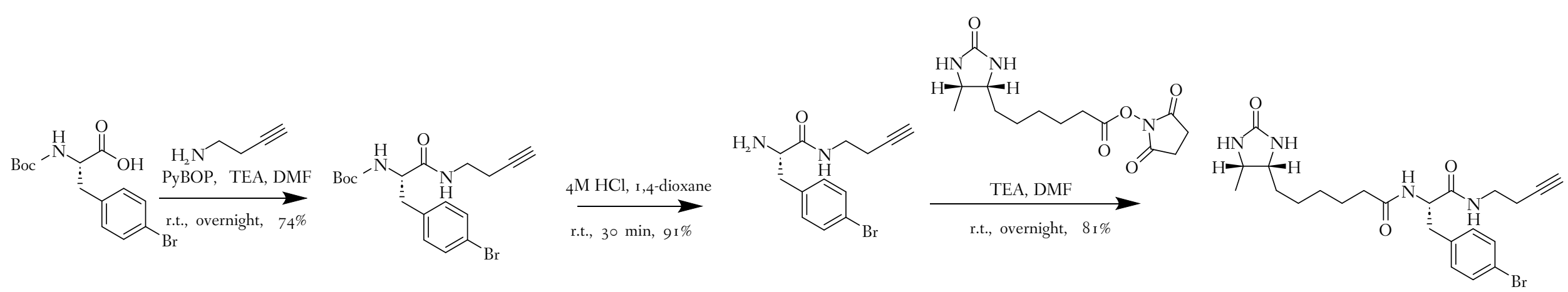

1 2a 3a 4a

Br-DTB- Alkyne

Step 1. tert-butyl (S)-(3-(4-bromophenyl)-1-(but-3-yn-1-ylamino)-1-oxopropan-2-yl)carbamate (2a)**:** To a stirred solution of acid **1** (1.0 gm, 2.94 mmol) in anhydrous DMF (5 mL) was added PyBOP (1.53 gm, 2.94 mmol, 1.0 equiv.), but-3-yn-1-amine (241 μL, 2.94 mmol, 1.0 equiv.) and DIPEA (401 ul, 2.94 mmol, 1.0 equiv.) at 0 °C. The reaction mixture was allowed to warm up to r.t. and stirred overnight for ~12 h. After completion of the reaction was monitored by TLC, and the Solvent was removed under reduced pressure and the mixture was added saturated Na_2CO_3 (20 mL) in H2O and stirred for 5 min. EtOAc was used for extraction (3 × 00 mL), the combined solvent was washed with brine (20 mL) and H_2O (20 mL) and dried over Na_2SO_4. The combined organic solution was subjected to flash chromatography under gradient conditions (0-40% EtOAc in hexane) to afford amide (850 mg, 974%) as a white solid ^{1}H NMR (400 MHz, $CDCl_3$) δ 7.41 (ddd, J = 8.3, 5.6, 1.9 Hz, 2H), 7.08 (dt, J = 8.4, 2.0 Hz, 2H), 6.46 (s, 1H), 5.23 (s, 1H), 4.33 (s, 1H), 3.33 (dp, J = 20.8, 6.9 Hz, 2H), 3.00 (d, J = 7.2 Hz, 2H), 2.36 – 2.24 (m, 2H), 1.95 (q, J = 1.9 Hz, 1H), 1.45 – 1.36 (m, 9H). ^{13}C NMR (100 MHz, $CDCl_3$) δ 224.60, 171.17, 157.72, 155.44, 135.72, 131.68, 131.05, 120.89, 81.06, 77.36, 77.04, 76.72, 70.16, 55.59, 38.03, 28.27, 19.22.

Step 2. **(S)-2-amino-3-(4-bromophenyl)-N-(but-3-yn-1-yl)propenamide** (3a)**:** Hydrochloric acid (4 M HCl in H_2O:1,4-dioxane (1:3, 4 mL)) was added dropwise to 2a (600 mg, 1.52 mmol) at 0 °C. The resulting mixture was

stirred for ~2 h at room temperature. After completion of the reaction, the reaction mixture Solvent was removed under reduced pressure, and the mixture was washed with diethyl ether (3 × 5 mL) and dried to afford amine **3** as an HCl salt as a white solid (410 mg, 1.04 mmol, 91%). The crude product was used for the next step without further purification. ^{1}H NMR (400 MHz, CD_3OD) δ 7.54 – 7.48 (m, 2H), 7.25 – 7.18 (m, 2H), 4.05 (t, J = 7.2 Hz, 1H), 3.43 – 3.20 (m, 2H), 3.19 – 3.02 (m, 2H), 2.34 – 2.32 (m, 1H), 2.32 – 2.29 (m, 2H). ^{13}C NMR (100 MHz, CD_3OD) δ 170.30, 168.09, 133.46, 131.73, 131.10, 121.33, 80.52, 69.72, 54.02, 38.27, 36.63, 18.20.

Step 3. N-((S)-3-(4-bromophenyl)-1-(but-3-yn-1-ylamino)-1-oxopropan-2-yl)-6-((4R,5S)-5-methyl-2-oxoimidazolidin-4-yl)hexanamide (4a)**:** To a stirred solution of salt **3a** (500 mg, 1.51 mmol) in anhydrous DMF (10 mL) was added TEA (412 μL, 2.30 mmol, 2.0 equiv.) and desthiobiotin NHS ester (324 mg, 1.15 mmol, 1.0 equiv.) at 0 °C. The reaction mixture was allowed to warm up to r.t. and stirred overnight for ~12 h. Solvent was removed under reduced pressure, and the mixture was subjected to flash chromatography under gradient conditions (0-5% MeOH in DCM). Fractions containing the product were combined, dried, and subjected to flash chromatography (C18 column) under gradient conditions (20-100% MeOH in H_2O) to afford product (680 mg, 1.38 mmol, 81%) as a white solid ^{1}H NMR (400 MHz, $CDCl_3$) δ 7.52 (d, J = 5.9 Hz, 1H), 7.38 (dd, J = 8.5, 2.0 Hz, 2H), 7.12 – 7.07 (m, 2H), 6.78 (s, 1H), 5.79 (s, 2H), 4.71 (q, J = 8.3 Hz, 1H), 3.91 (t, J = 7.0 Hz, 1H), 3.75 (q, J = 5.6 Hz, 1H), 3.39 (dq, J = 12.9, 6.5 Hz, 1H), 3.27 (dt, J = 13.1, 6.5 Hz, 1H), 2.98 (qd, J = 13.8, 7.3 Hz, 2H), 2.31 (dp, J = 6.8, 3.4 Hz, 2H), 2.18 (t, J = 7.4 Hz, 2H), 1.97 (t, J = 2.6 Hz, 1H), 1.58 (dq, J = 31.8, 6.7 Hz, 2H), 1.47 – 1.37 (m, 3H), 1.25 (s, 1H), 1.18 (d, J = 6.5 Hz, 5H). ^{13}C NMR (100 MHz, $CDCl_3$) δ 174.27, 173.59, 171.65, 157.81, 135.79, 131.59, 130.99, 120.77, 70.15, 56.45, 54.20, 51.85, 38.14, 37.56, 35.23, 29.39, 28.00, 25.78, 25.06, 19.13, 15.71. MS (ESI$^+$) *m/z* calc'd for $C_{23}H_{31}BrN_4O_3$ $[M+H]^+$: 491.15 found 491.20.

3. NMR(^{1}H/^{13}C) of compound (2)

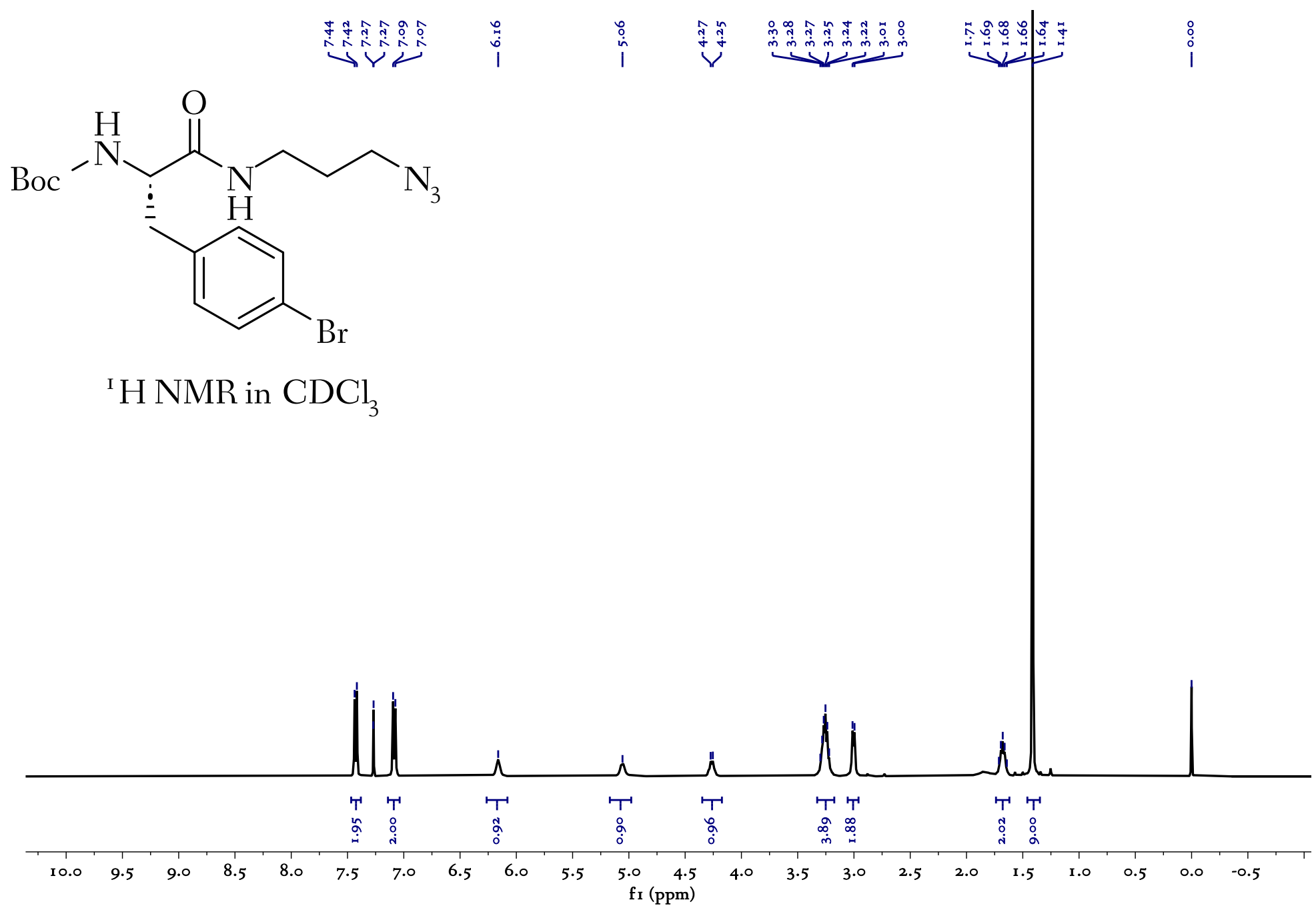

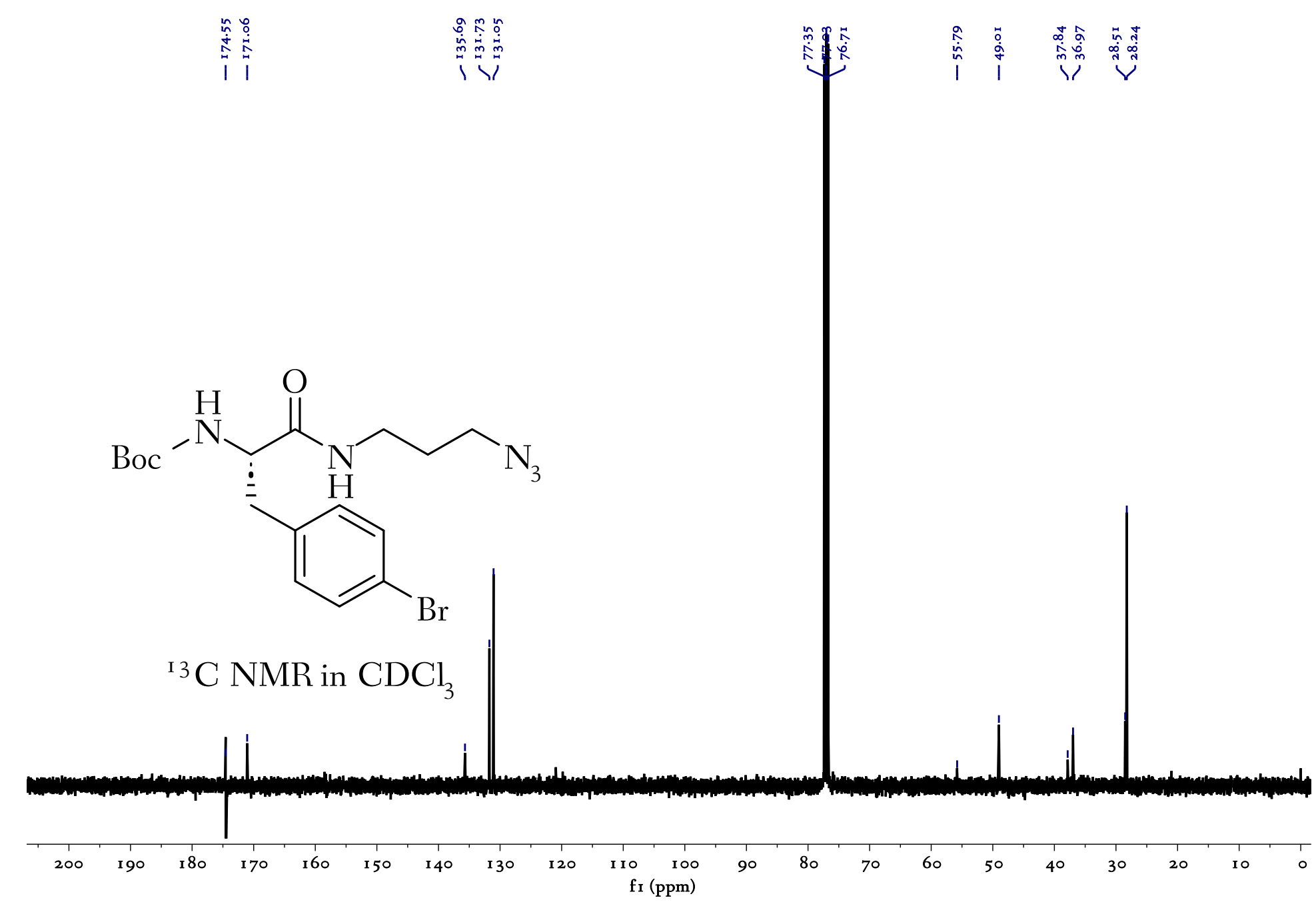

Figure S1. ^{1}H-/ ^{13}C-NMR spectra of (**2**) in $CDCl_3$.

4. NMR(^{1}H/^{13}C) of compound (3)

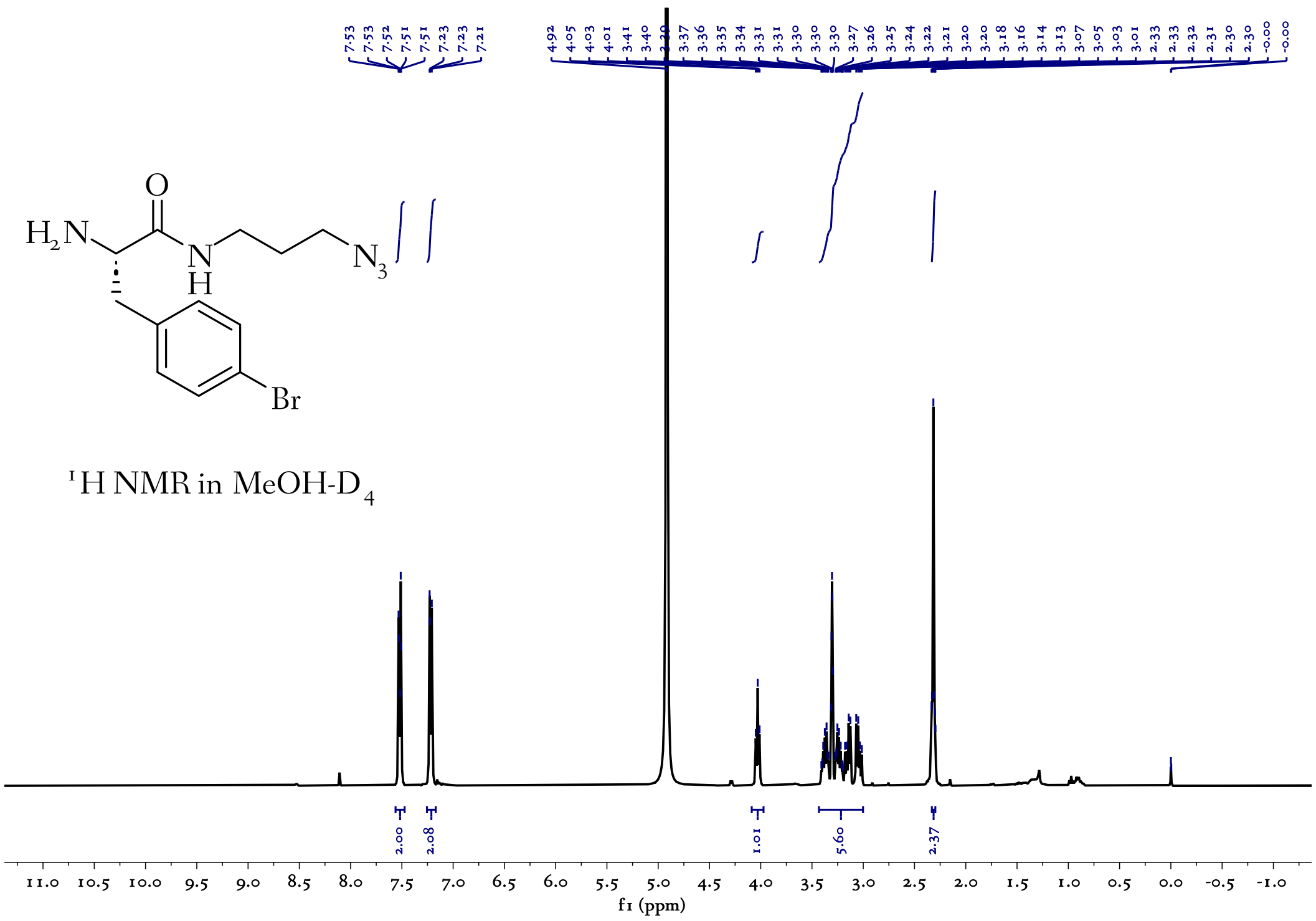

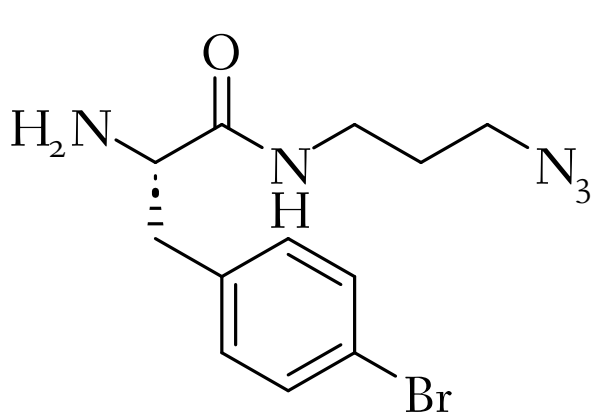

^{13}C NMR in MeOH-D$_4$

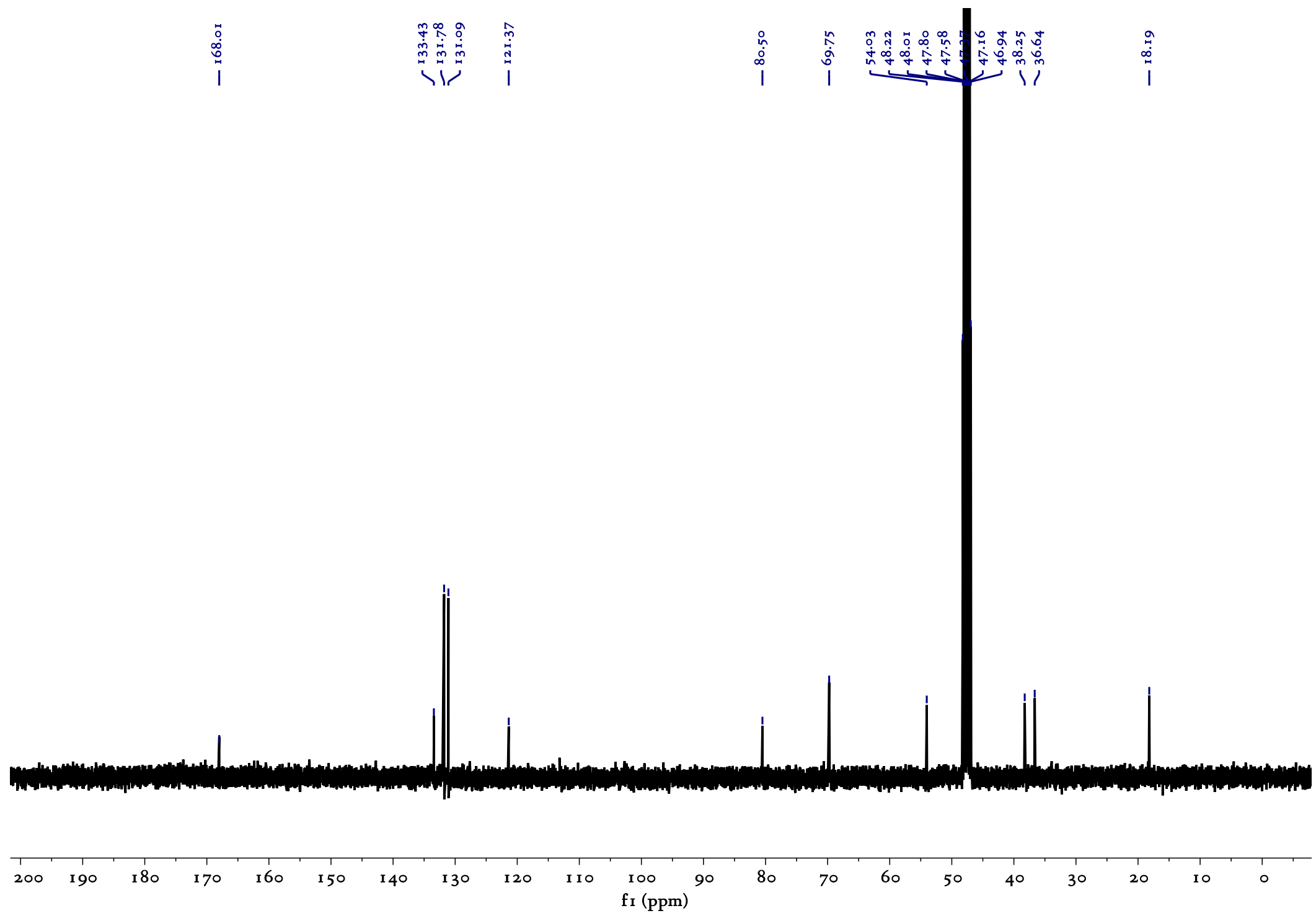

Figure S2. ^{1}H-/ ^{13}C-NMR spectra of (**3**) in CD_3OD.

5. NMR(^{1}H/^{13}C) of compound (4)

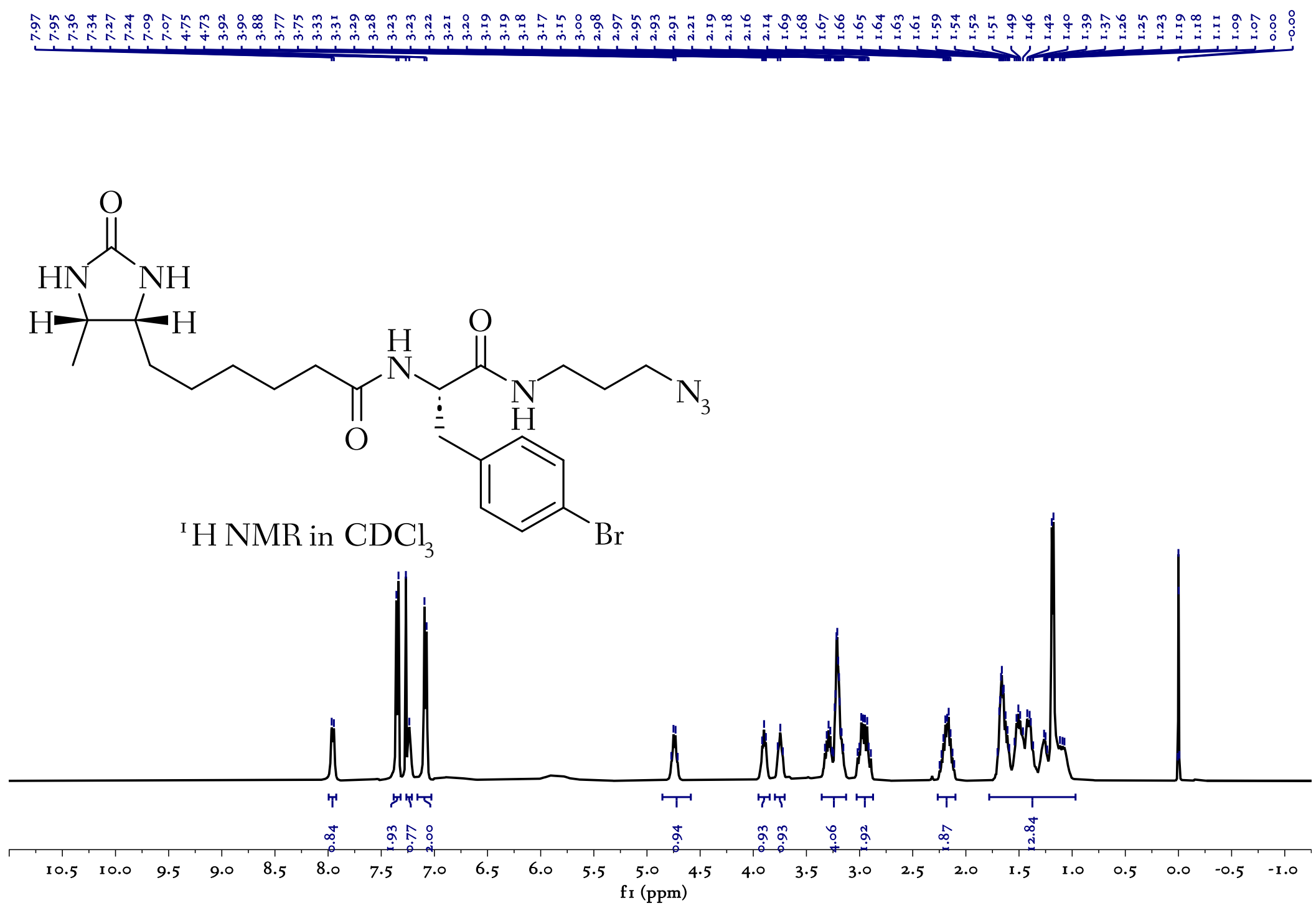

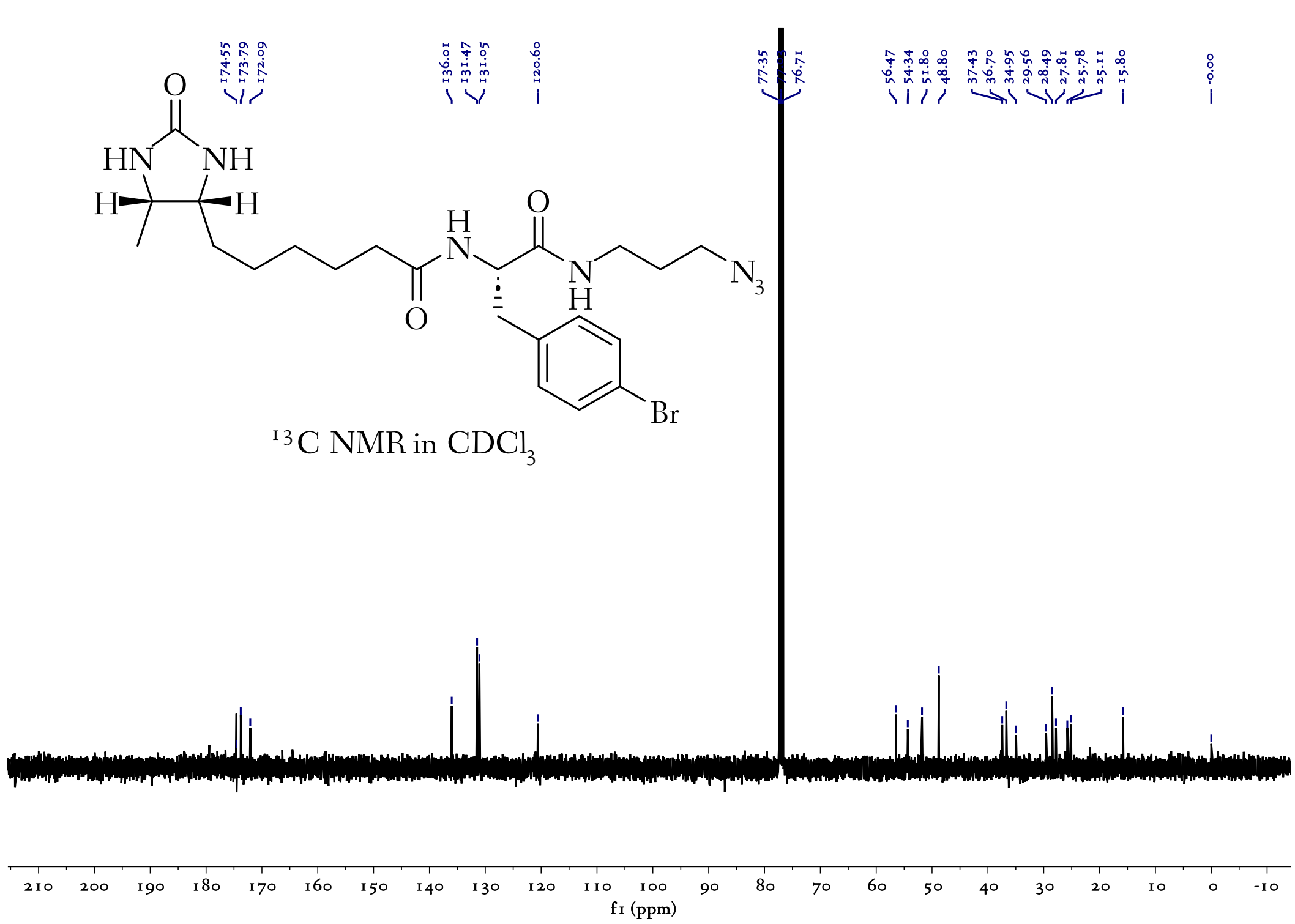

Figure S3. ^{1}H-/ ^{13}C-NMR spectra of (4) in $CDCl_3$.

6. ESI/MS Spectra of compound (4)

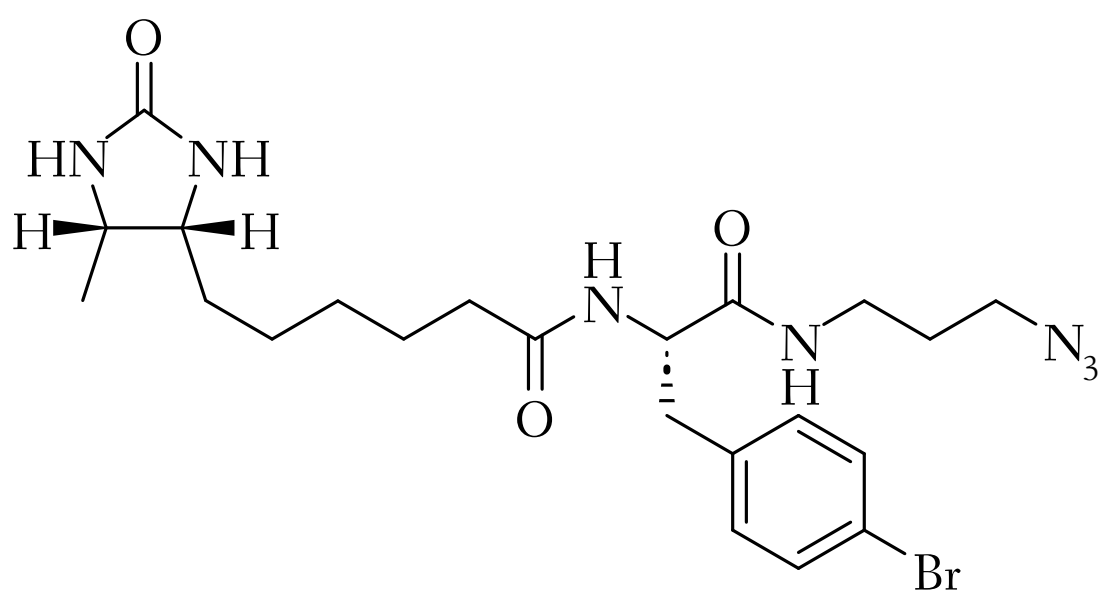

Chemical Formula: $C_{22}H_{32}BrN_7O_3$

Exact Mass: 521.17500

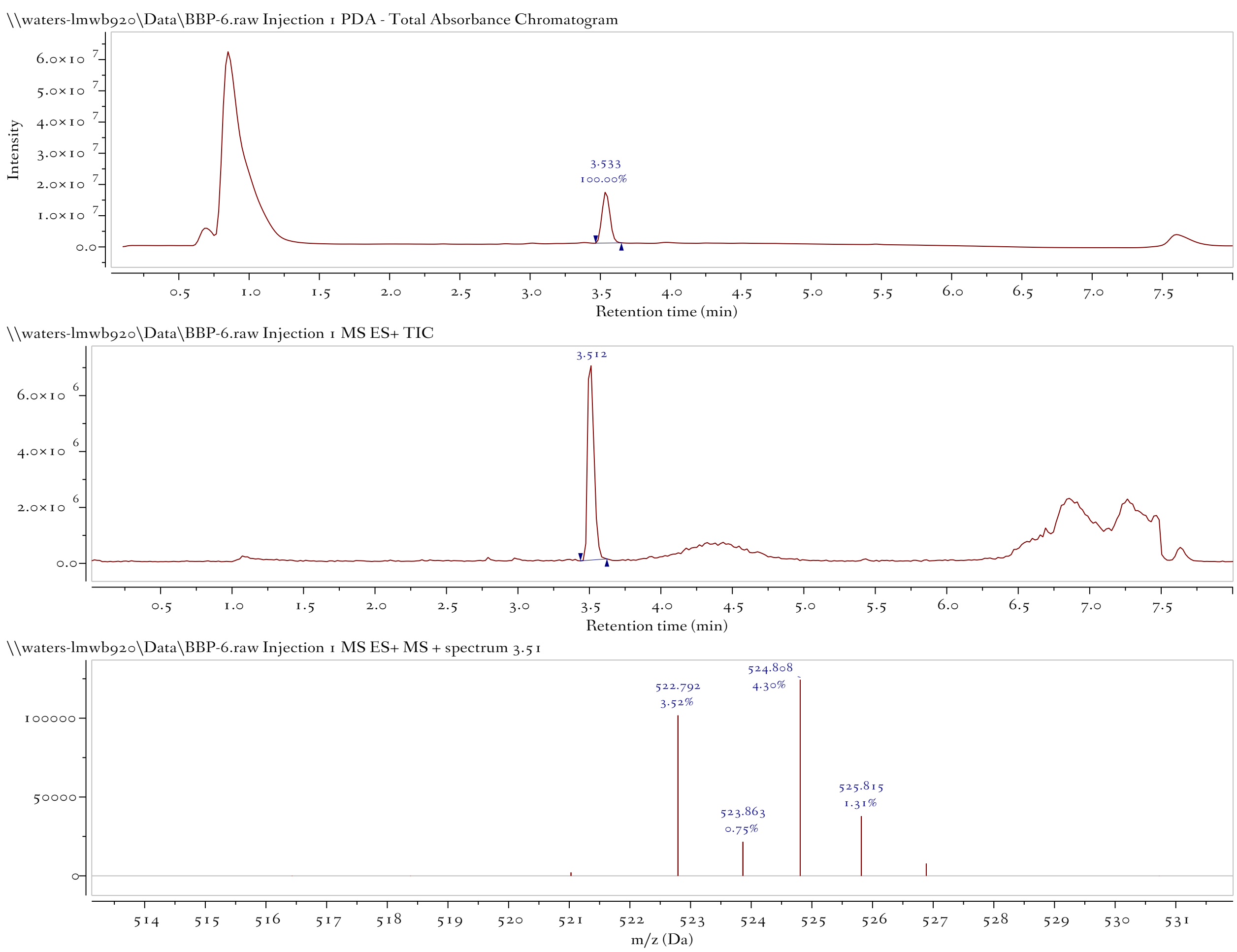

Figure S4. LC-MS spectra of compound (4).

7. NMR(^{1}H/^{13}C) of compound (2a)

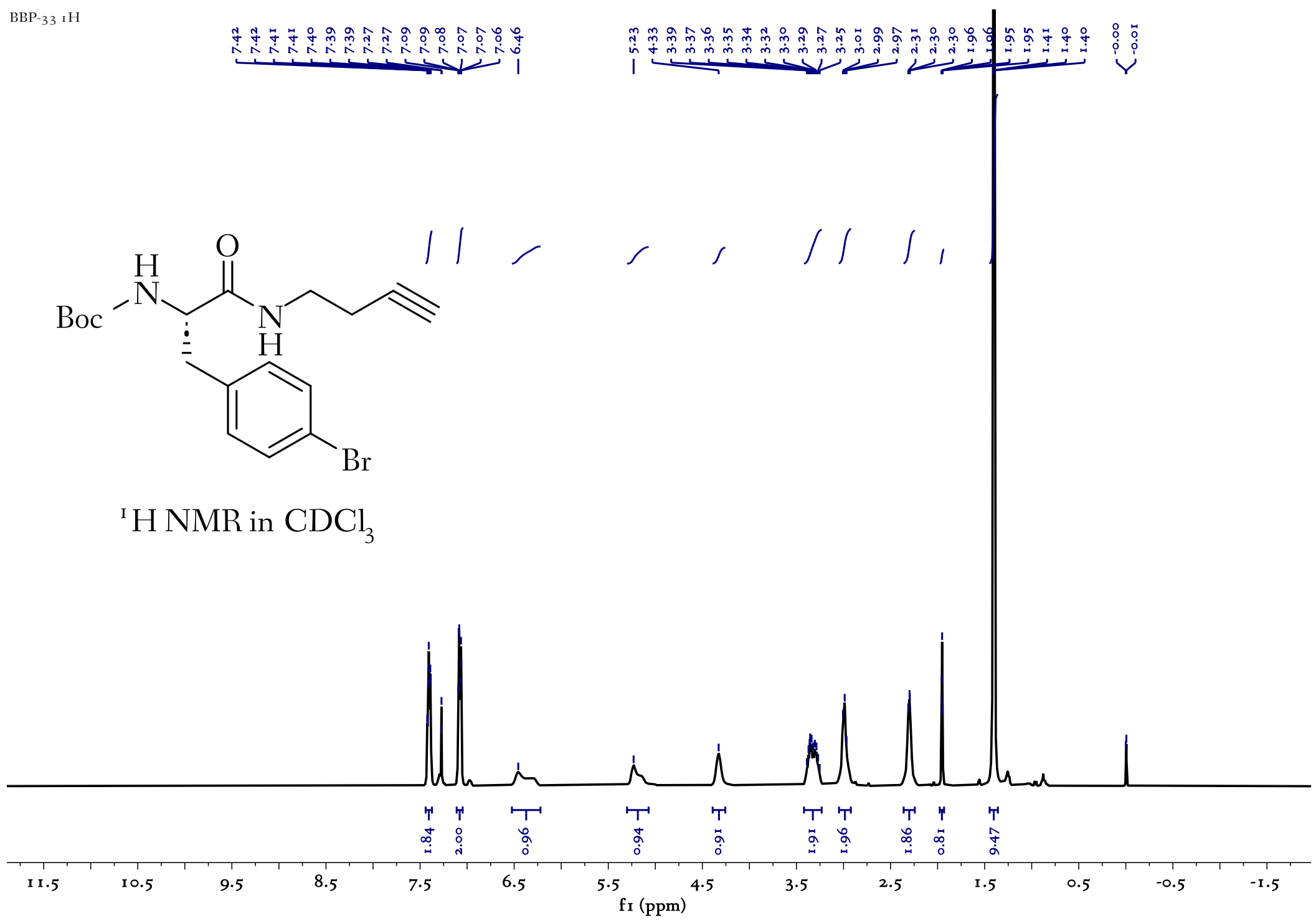

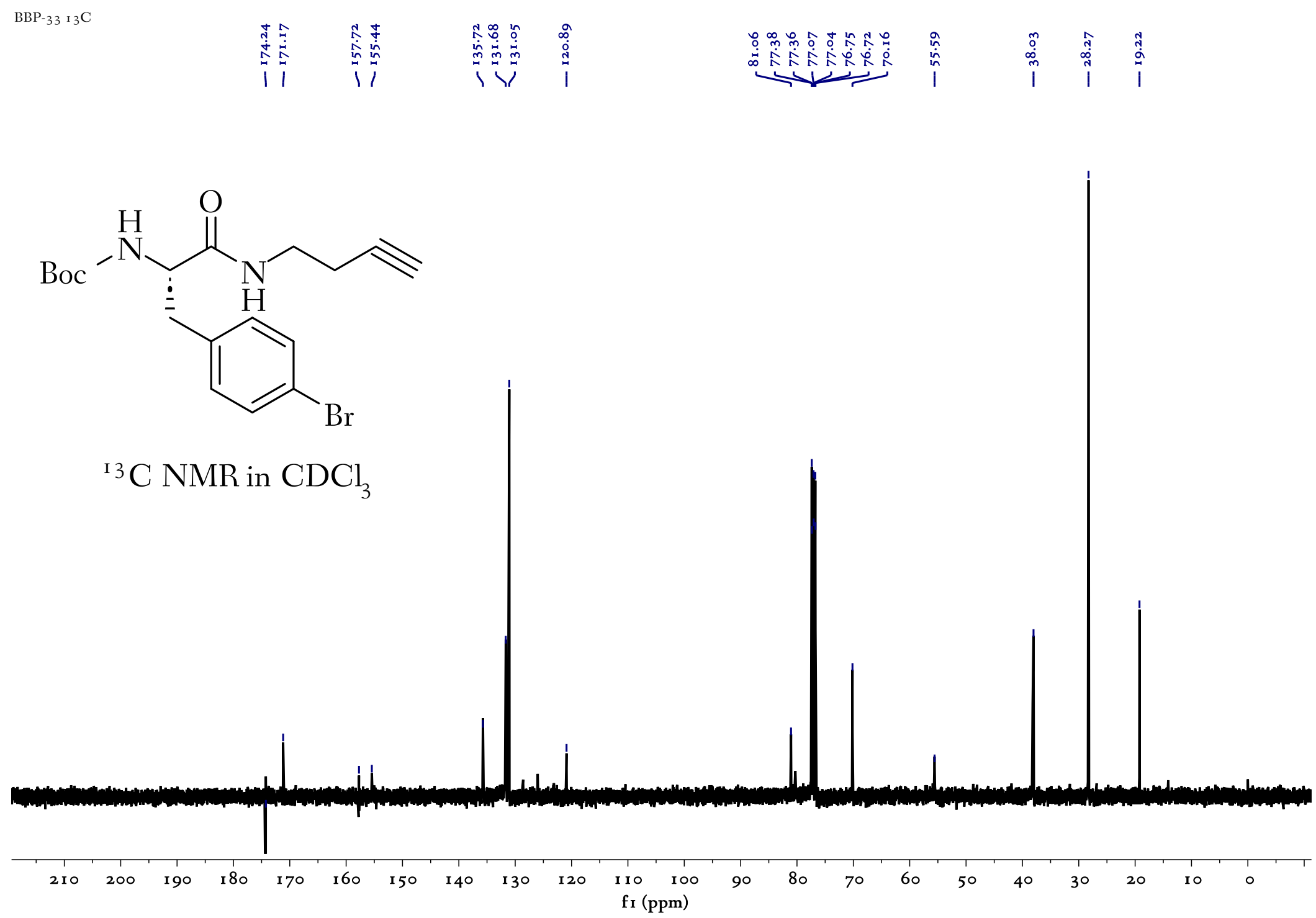

Figure S5. ^{1}H-/ ^{13}C-NMR spectra of (**2a**) in $CDCl_3$.

8. **NMR(^{1}H/^{13}C) of compound (3a)**

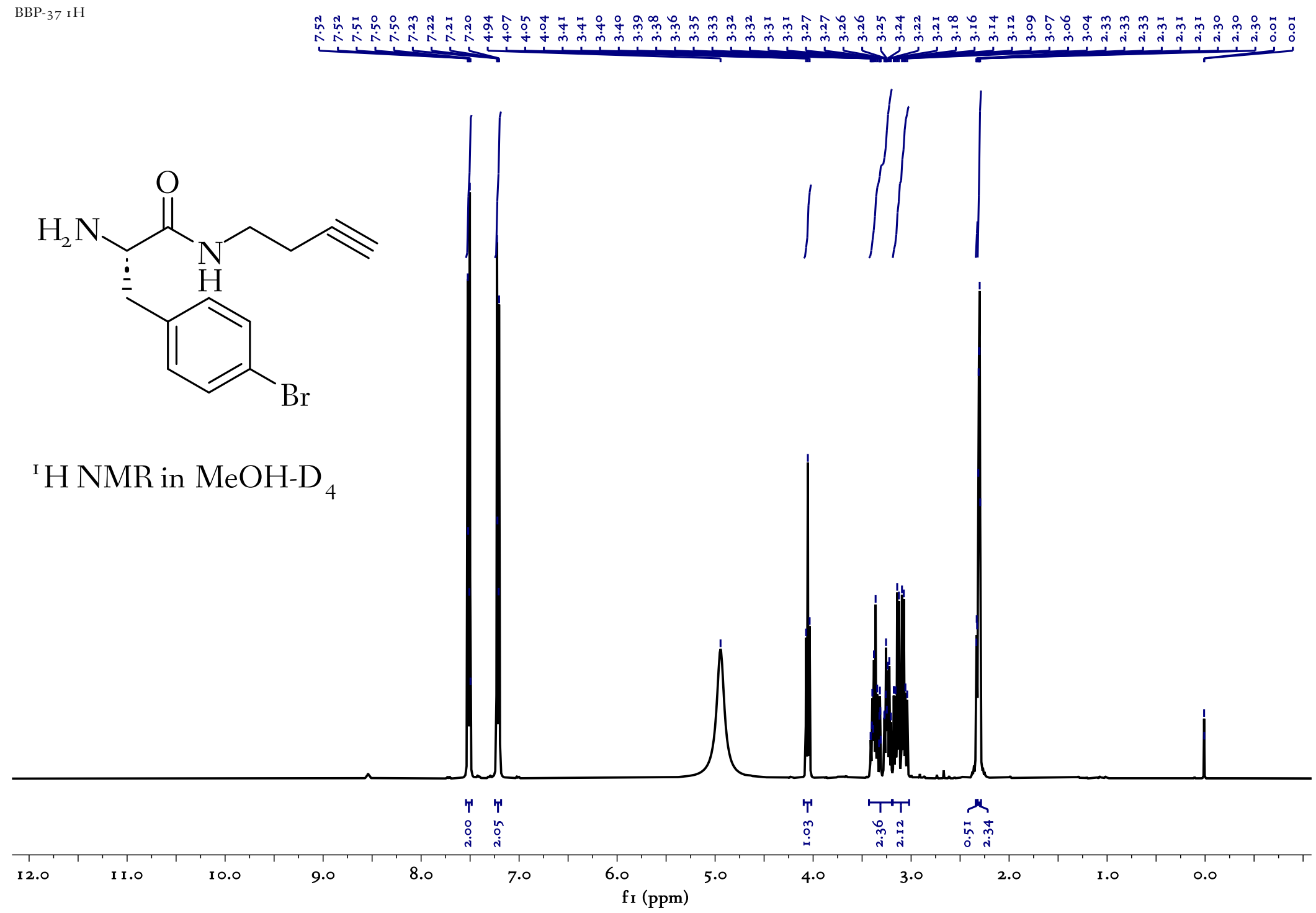
BBP-37 1H
H2N
O
N
H
Br
1H NMR in MeOH-D4
f1 (ppm)

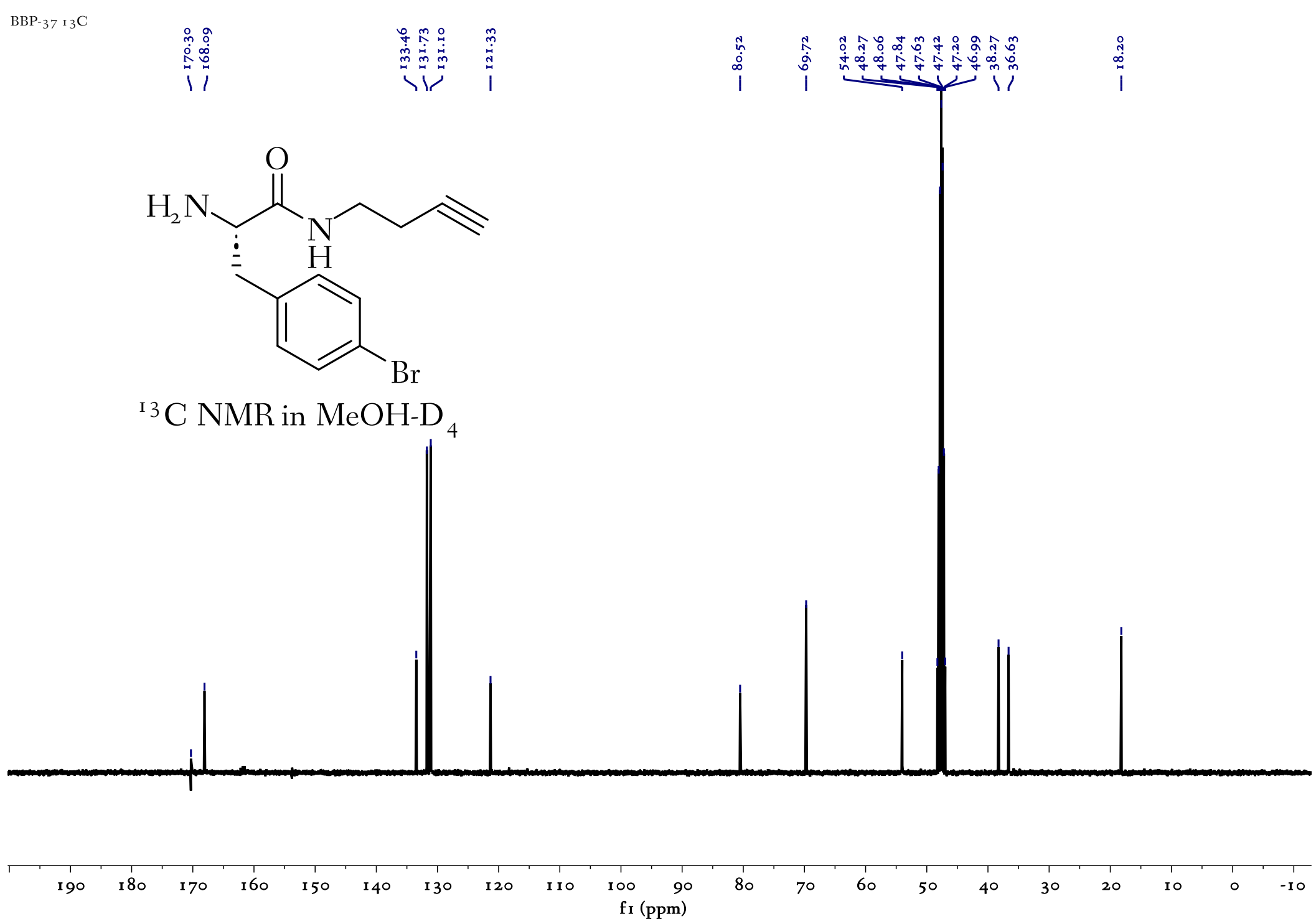
BBP-37 13C
170.30
168.09
133.46
131.73
131.10
121.33
80.52
69.72
54.02
48.27
48.06
47.84
47.63
47.42
47.20
46.99
38.27
36.63
18.20
H2N
O
N
H
Br
13C NMR in MeOH-D4
f1 (ppm)

Figure S6. ^{1}H-/ ^{13}C-NMR spectra of (**3a**) in CD_3OD.

9. NMR(^{1}H/^{13}C) of compound (3a)

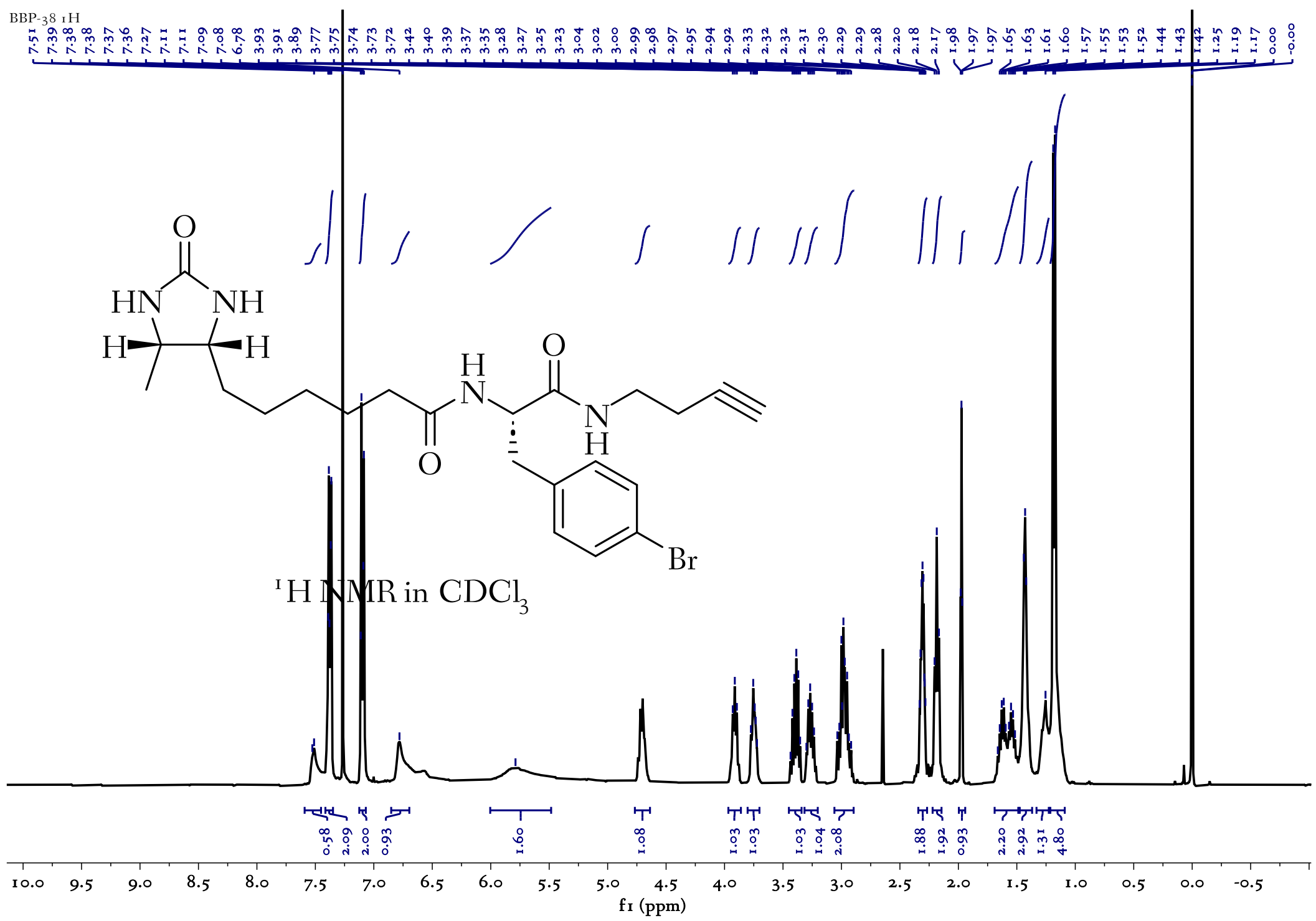

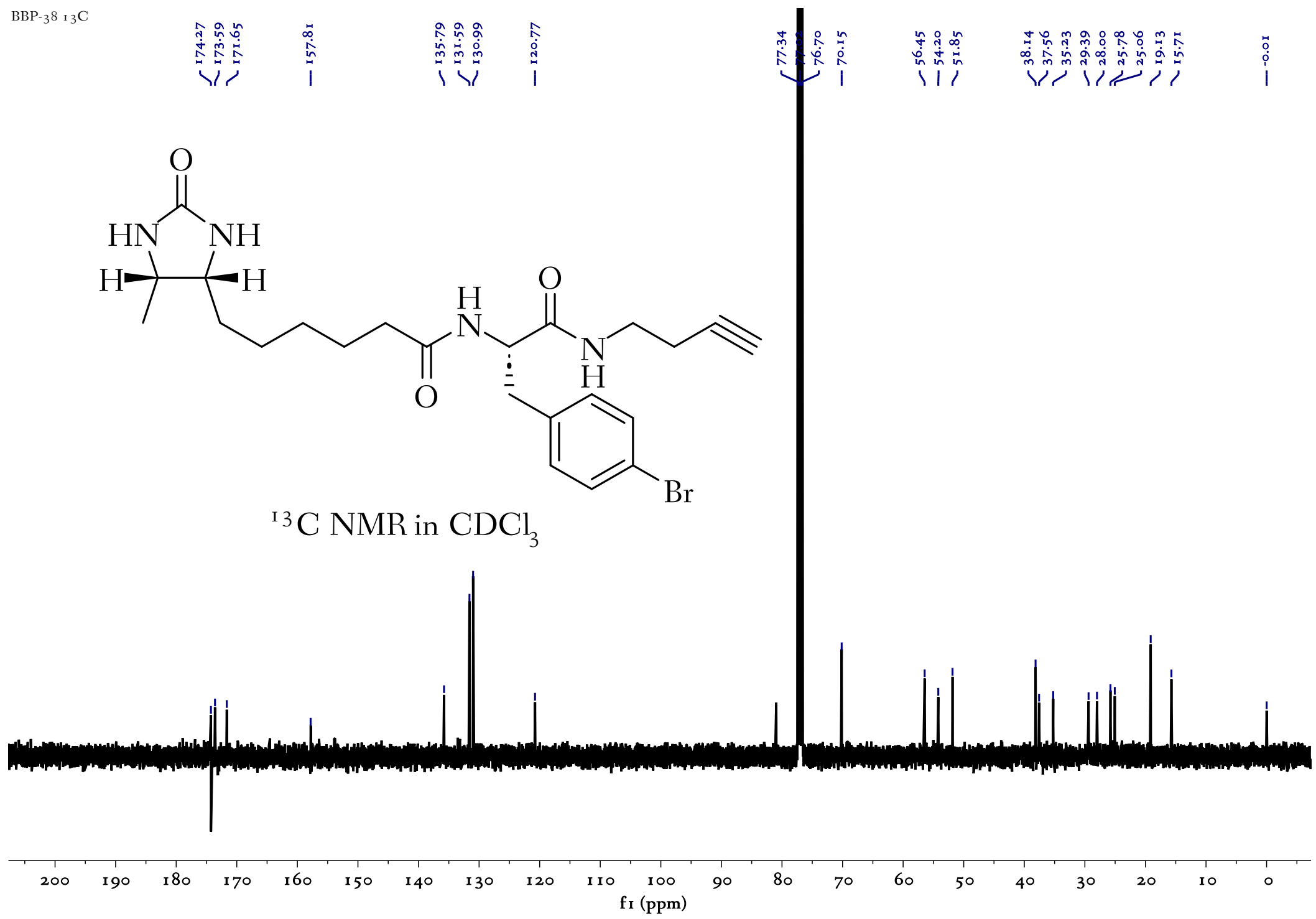

Figure S7. ^{1}H-/ ^{13}C-NMR spectra of (**4a**) in $CDCl_3$.

10. ESI/MS Spectra of compound (4a)

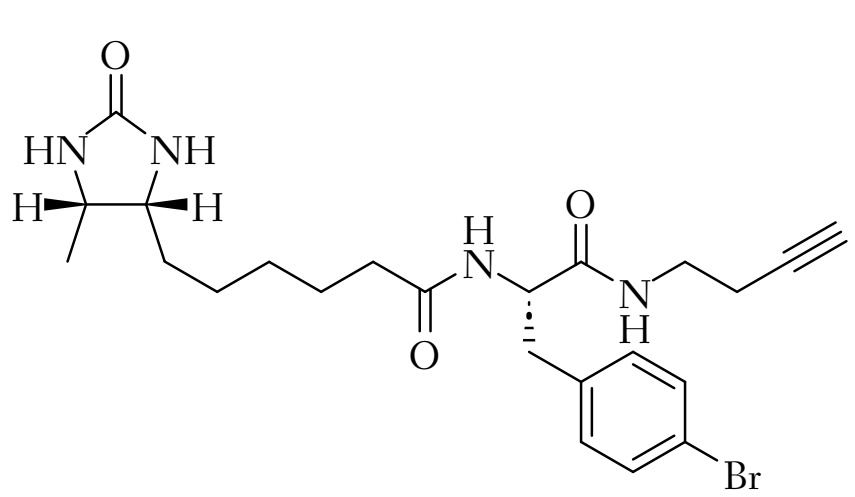

Chemical Formula: $C_{23}H_{31}BrN_4O_3$
Exact Mass: 490.15795

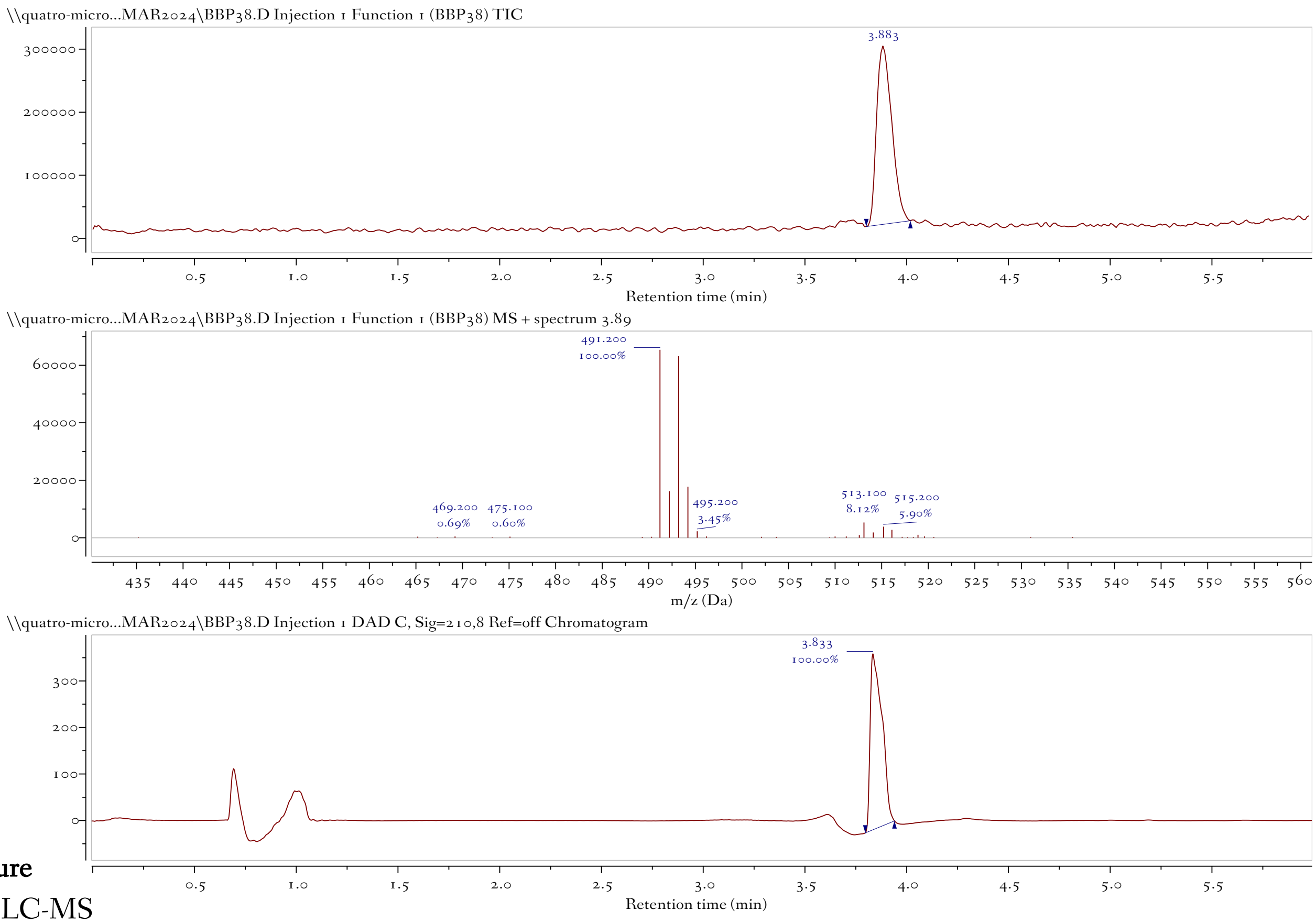

Figure S8. LC-MS spectra of compound (**4a**).

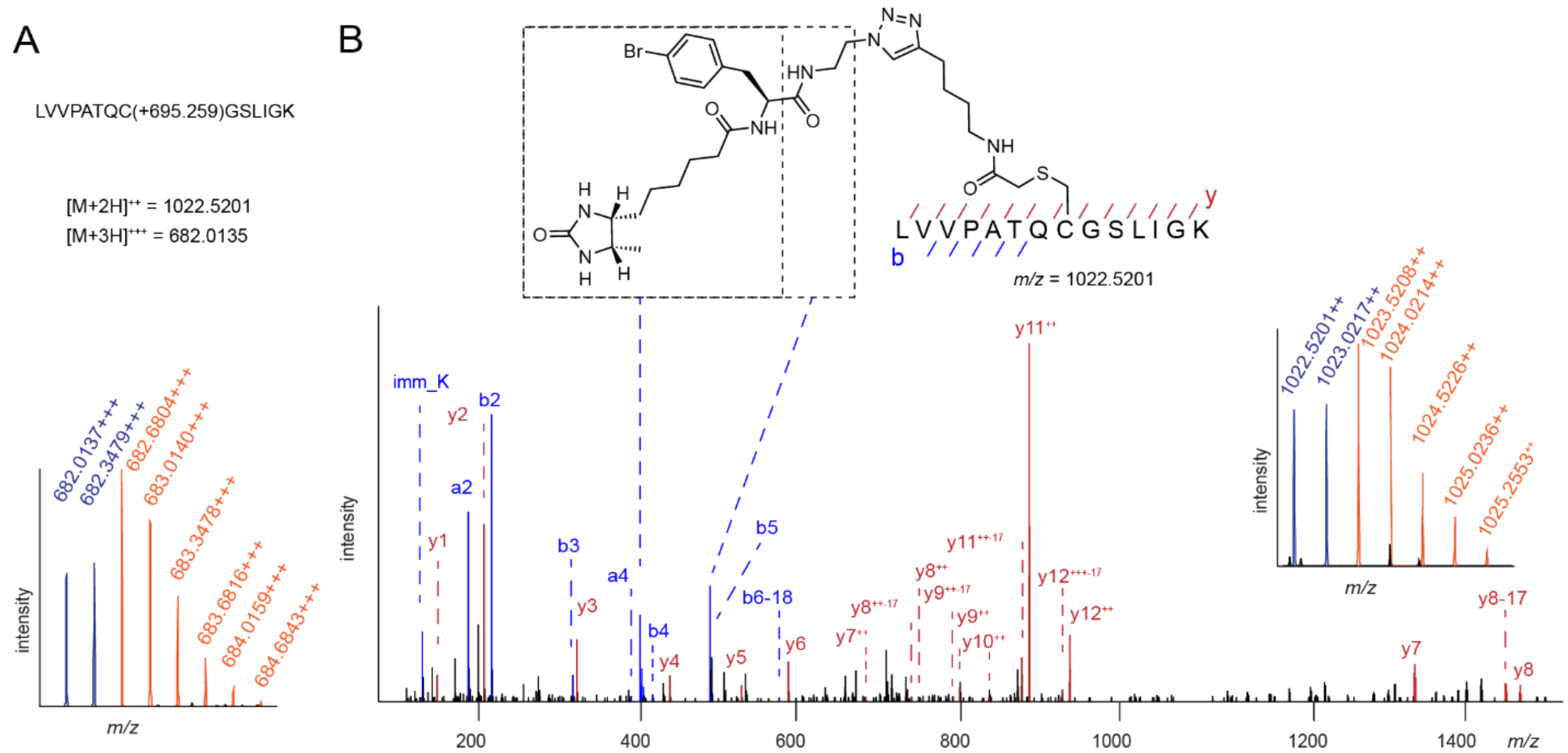

Figure S9. MS1 and MS2 mass spectrometry of a representative peptide. (A) Representative BrDTB peptide LVVPATQCC[+658.259]GSLIGK, which has z=3 in MS1. (B) The MS1 precursor and MS2 spectra of the peptide.

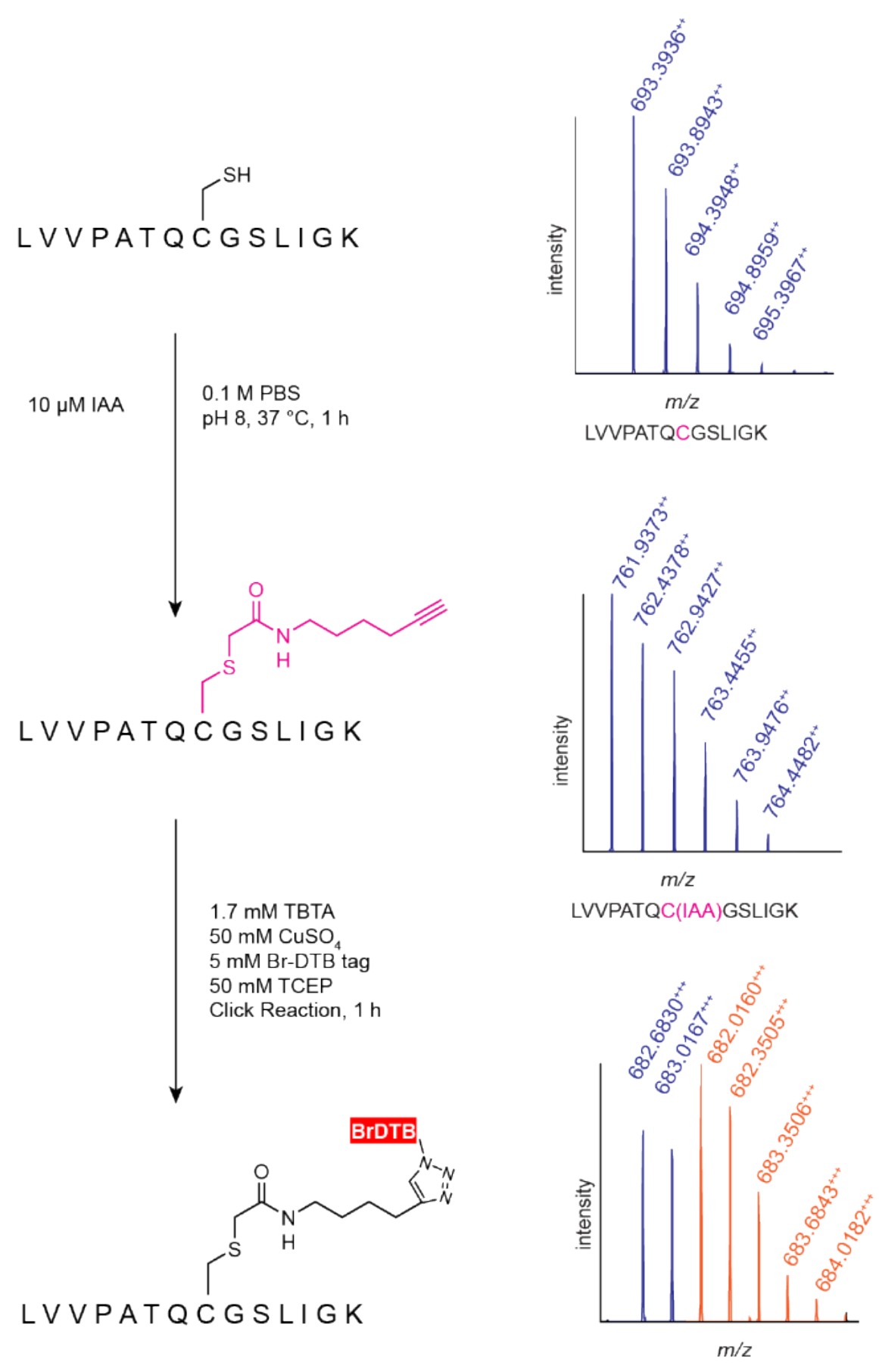

Figure S10. Synthetic peptide labeled with IAA followed by click chemistry with Br-DTB. Reaction conditions: 10 μg of synthetic peptide (LVVPATQCGSLIGK) and 10 μM iodoacetamide-alkyne (IAA) were incubated in 0.1 M PBS, pH 8, at 37°C for 1 h. In the second step, the click reaction included Br-DTB Azide (5.0 mM), TBTA (1.7 mM), $CuSO_4$ (0.1 mM), and TCEP (50 mM).

11. Synthetic peptide labeled BrDTB and its MS1 and MS2 spectra

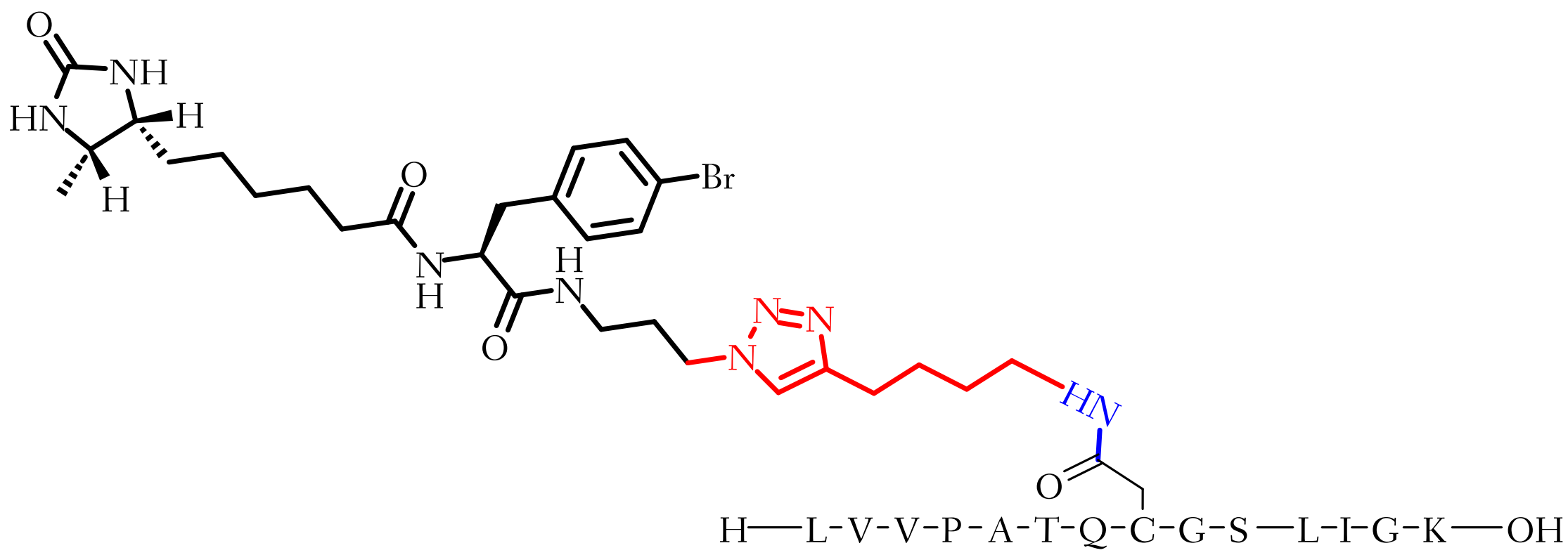

Chemical Formula: $C_{91}H_{151}BrN_{24}O_{22}S$

Exact Mass: 2043.03389

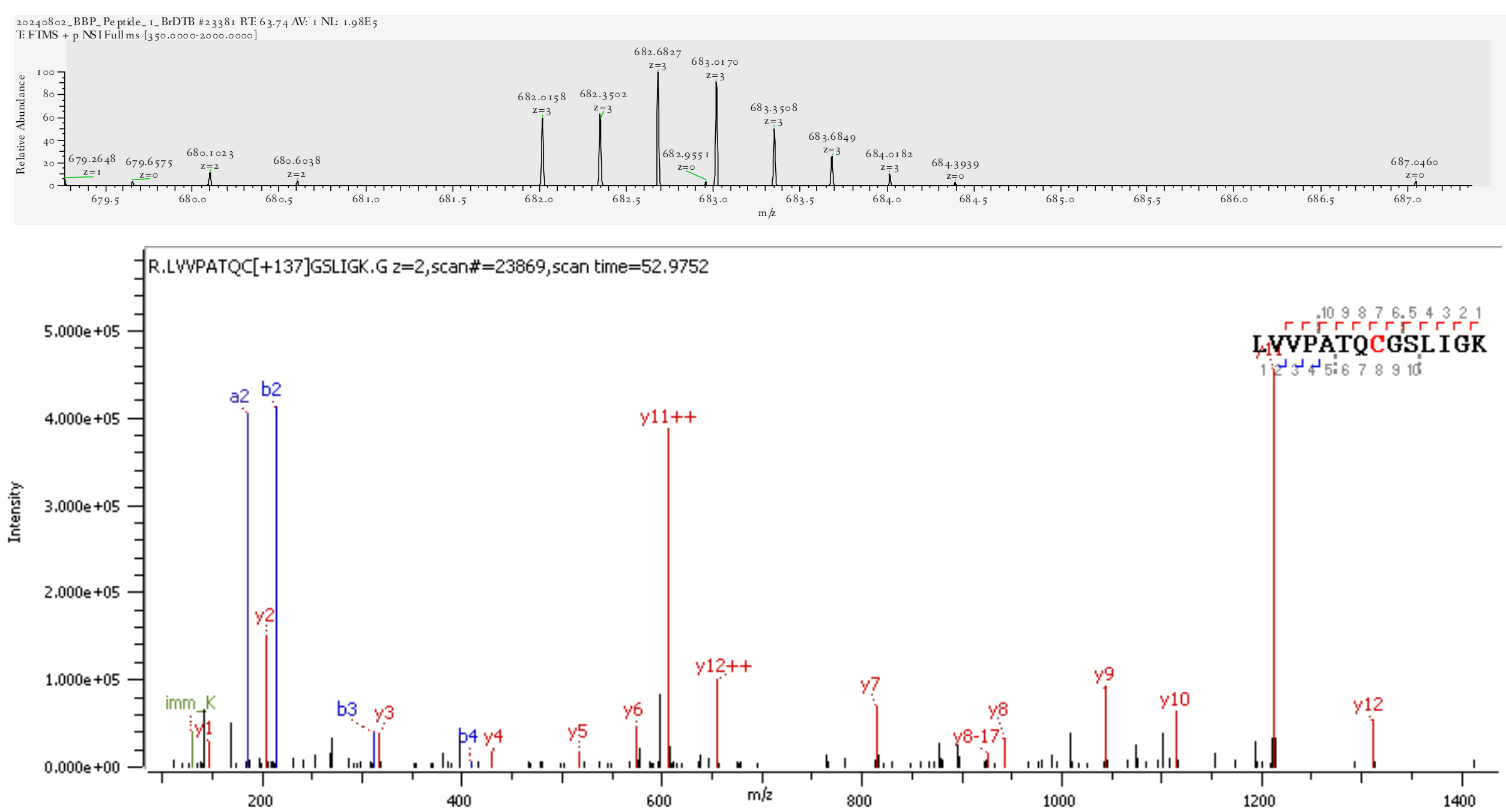

Figure S11. MS1 and MS2 identification of the synthetic peptide LVVPATQC[+658.259]GSLIGK labeled BrDTB.

12. Establishment of in-house Br-Matcher software for Br-Isotopic distribution peptide analysis

Brominated peptides were identified using an in-house Python-based software tool (Br-Matcher), developed to evaluate bromine isotopic signatures in mass spectrometry data. The algorithm iteratively calculates similarity scores between experimental and theoretical isotope distributions for all candidate peptides, enabling confident detection of the characteristic bromine M/M+2 pattern. Peptides were filtered based on similarity score thresholds, yielding candidates with isotope ratios centered around expected theoretical values. MS data were processed using output files generated by Byonic. Raw mass spectrometry files (.raw) were converted to. mzXML format using RawConverter (version 1.1.0.23) with monoisotopic peak selection enabled. Br-Matcher was subsequently applied to analyze MS1 spectra corresponding to brominated peptide identifications.

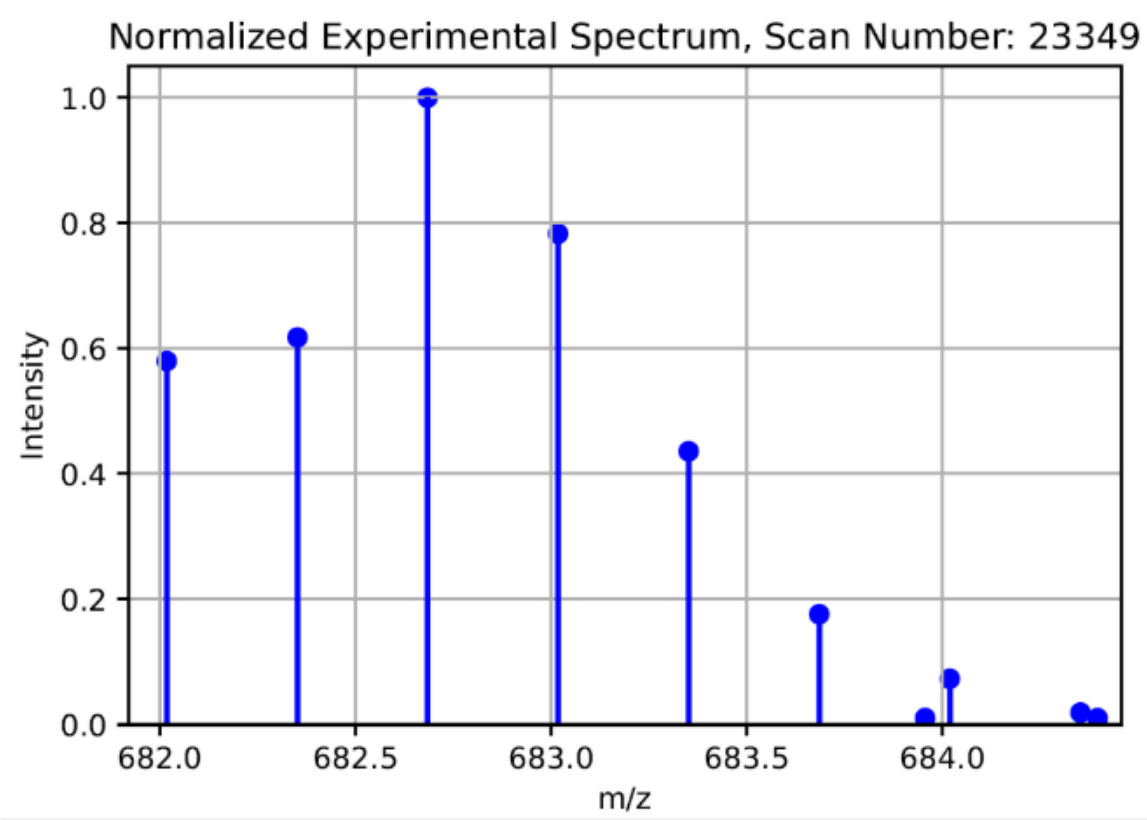

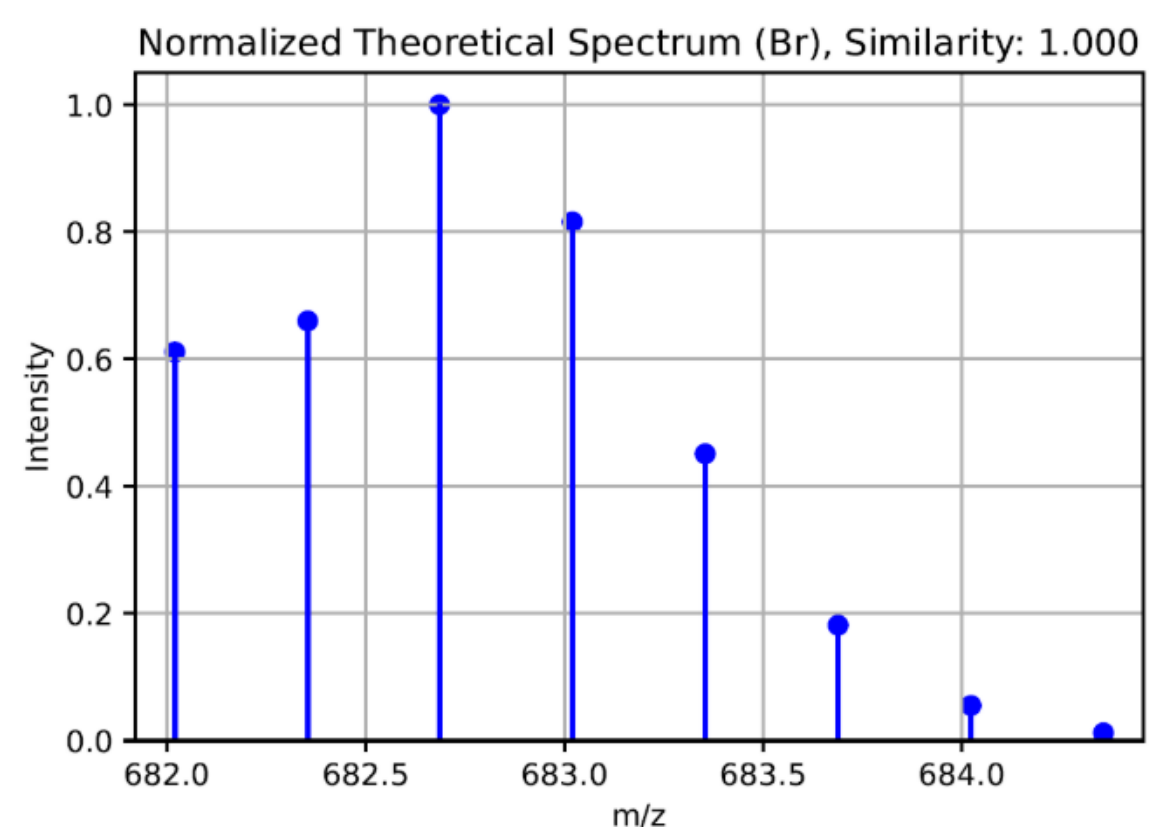

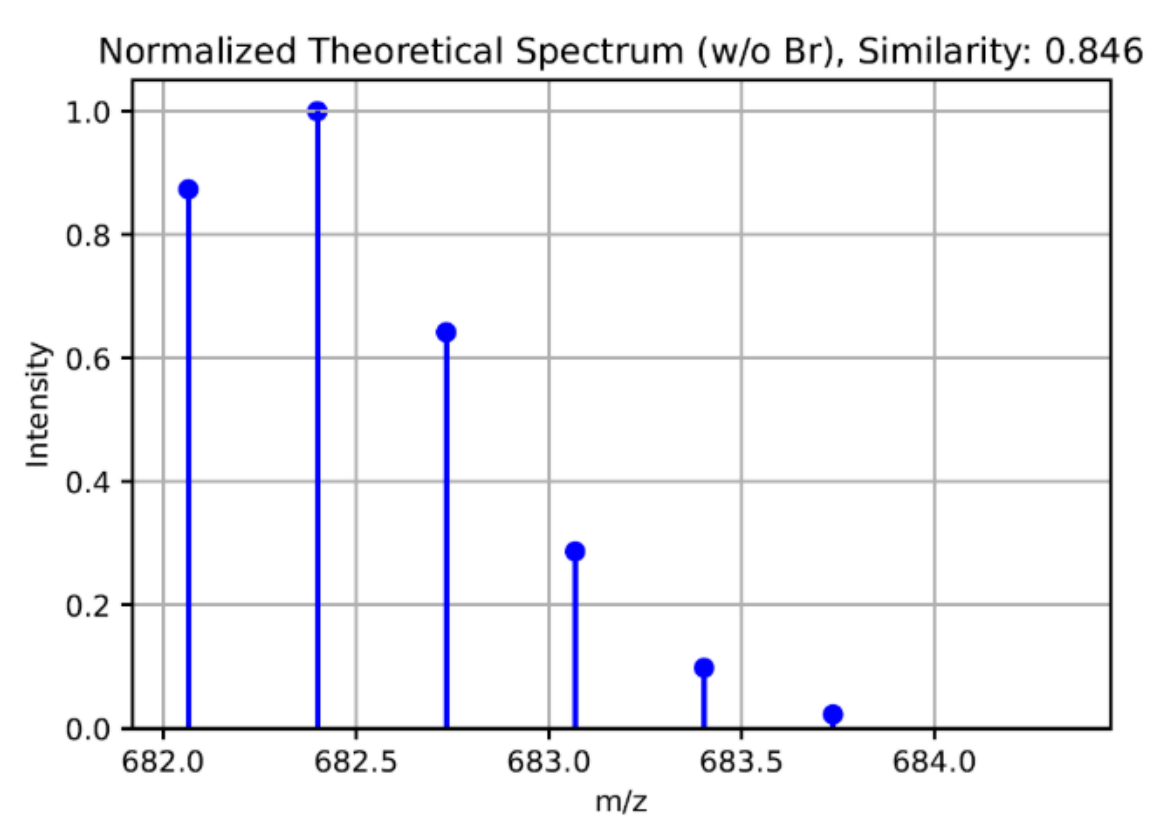

Figure S12. Representative peptide LVVPATQC[+658.259]GSLIGK after running Br-Matcher comparing the Experimental spectrum and the theoretical spectrum.

13. Example of an AHA-labeled BrDTB peptide and its MS1 and MS2 spectra

Peptide: SGSM[+485.172]DPSGAHPSVR

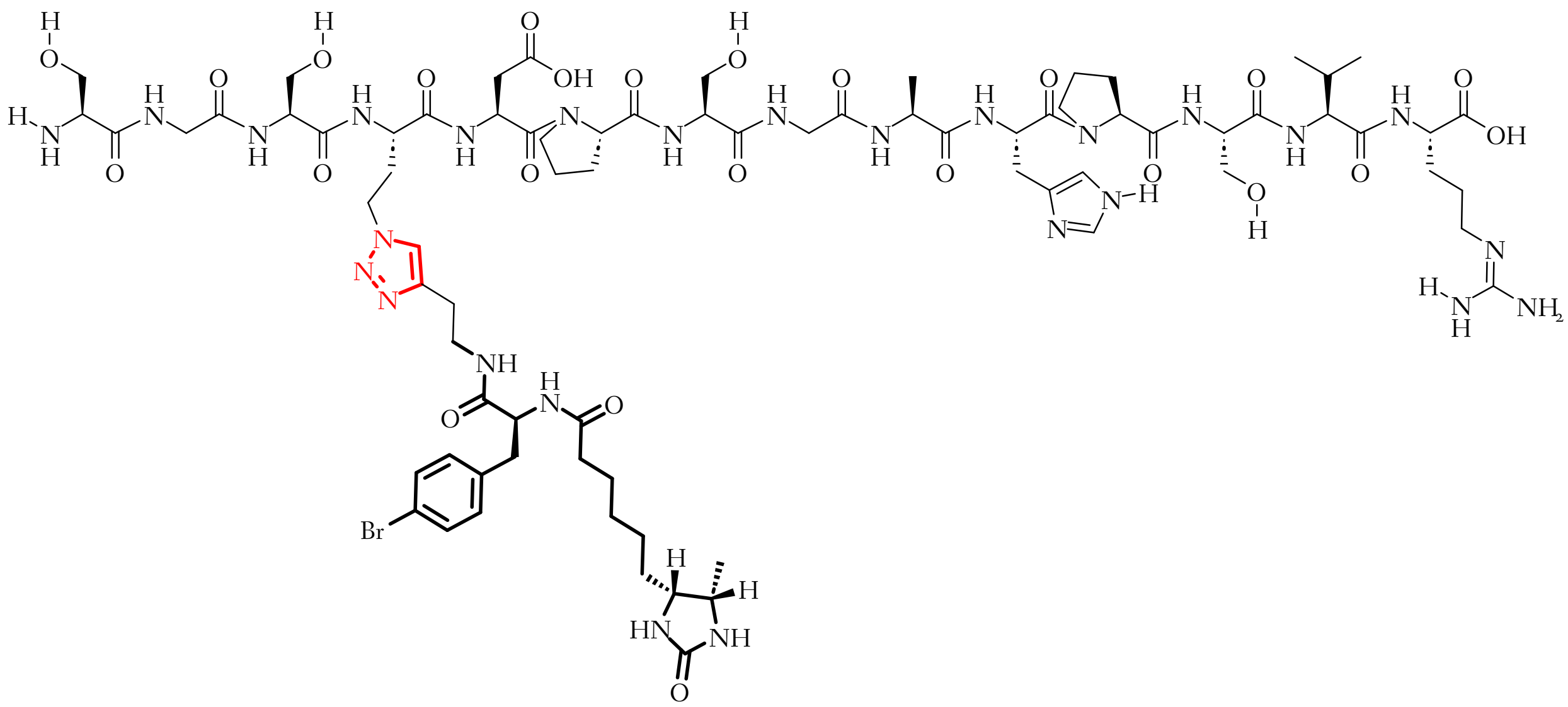

Chemical Formula: $C_{77}H_{117}BrN_{26}O_{24}$
Exact Mass: 1868.79174

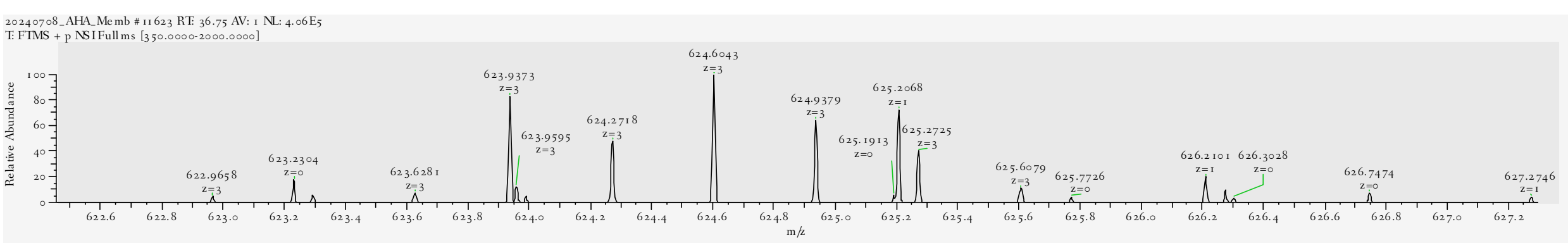

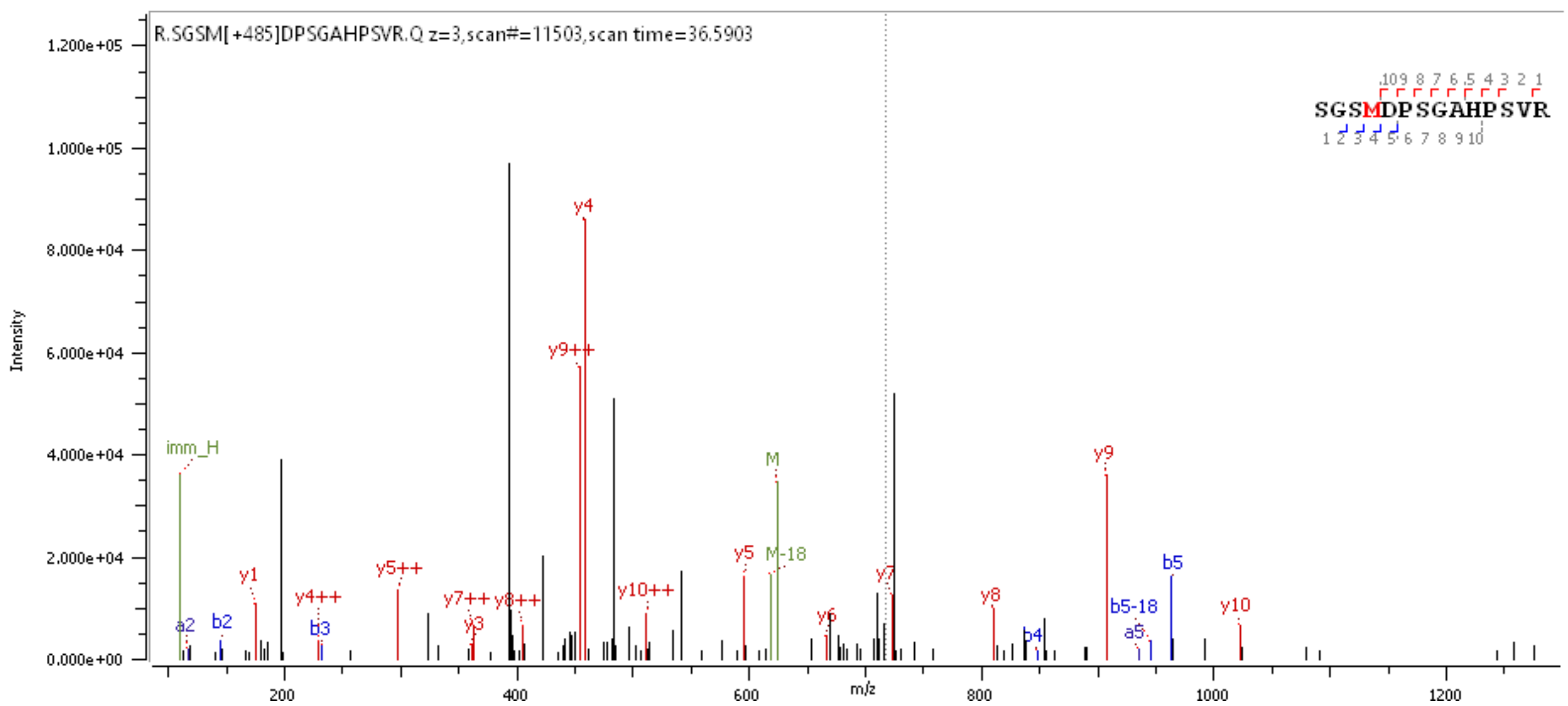
R.SGSM[+485]DPSGAHPSVR.Q z=3,scan#=11503,scan time=36.5903
SGSMDPSGAHPSVR
Intensity
m/z

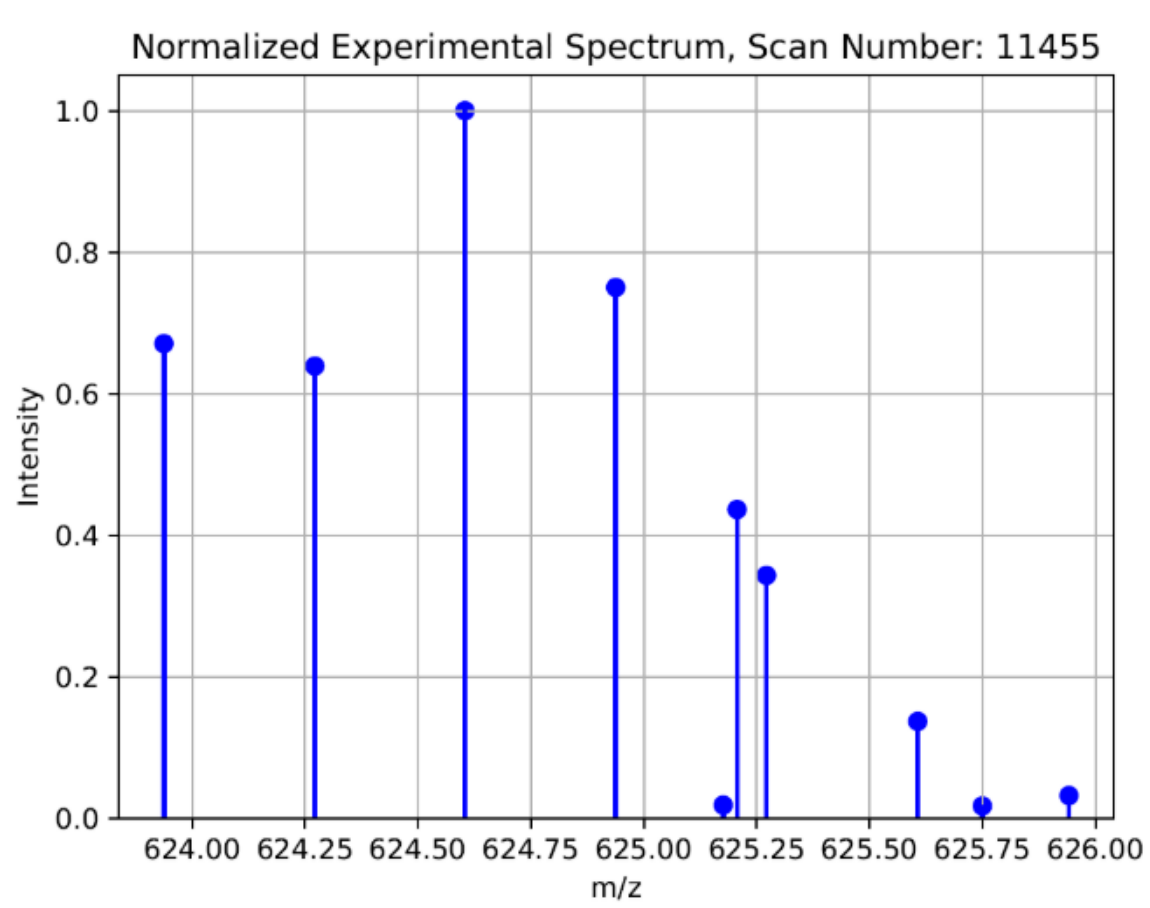
Normalized Experimental Spectrum, Scan Number: 11455
Intensity
m/z

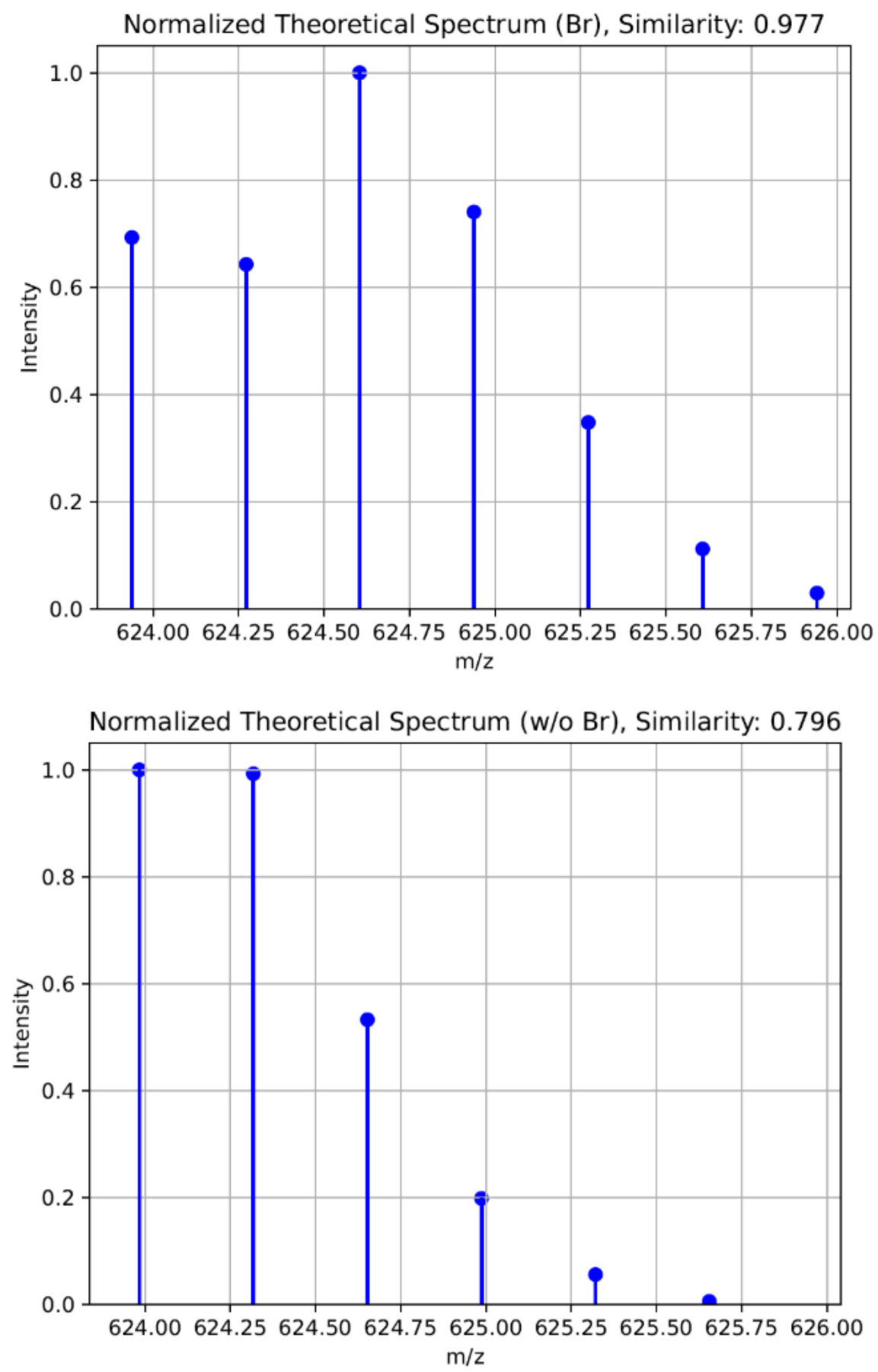

Figure S13. MS1 and MS2 identification of AHA-labeled BrDTB peptide and its similarity check.

14. Representative MS1 and MS2 spectra of PL-alkyne modified BrDTB peptide

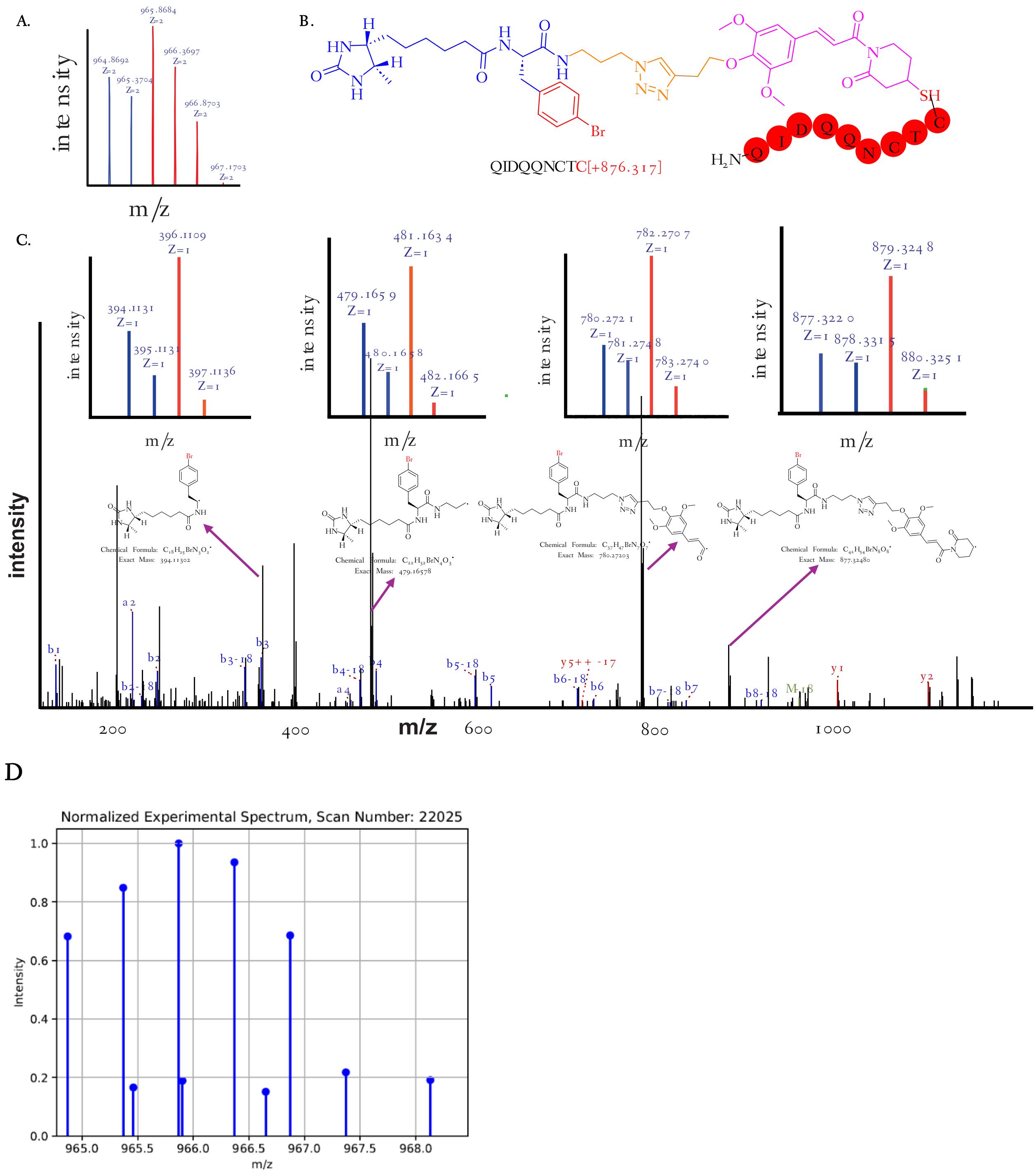

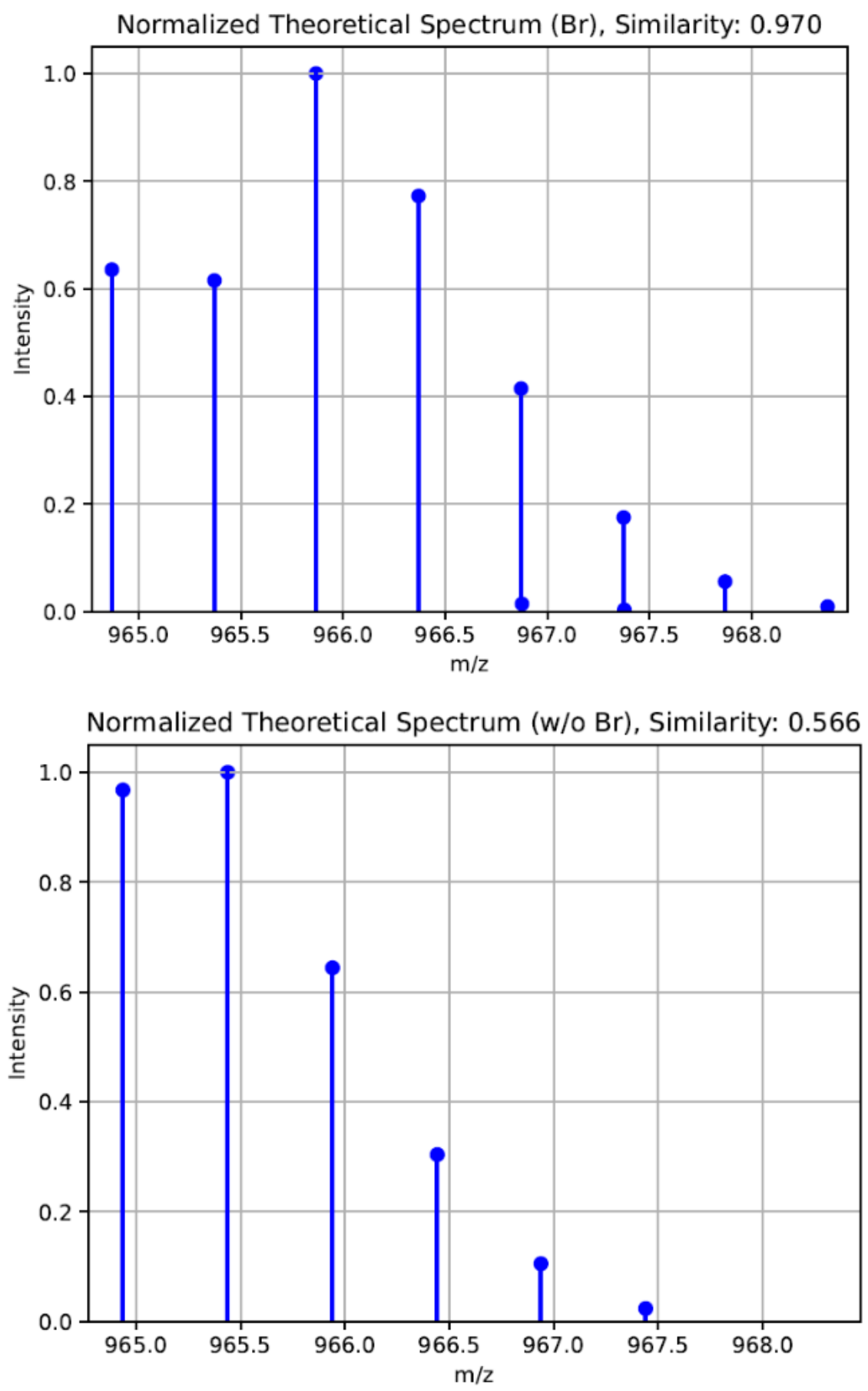

Figure S14. Representative MS1, MS2 spectra, and similarity check. (A) PL-alkyne modified BrDTB peptide QIDQQNCTC[+876.317] of precursor ion at m/z 694.86, which has Z=2, (B) Chemical structure of PL probe conjugated BrDTB cysteine peptides, (C) MS/MS spectra of PL-alkyne modified KEAP1 peptide QIDQQNCTC[+876.317] with bromo signature ions in fragmented form, (D) similarity check results by BrMatcher.

15. The KEAP1–CUL3–RBX1 dimer complex structure binds to the C-terminus of KEAP1

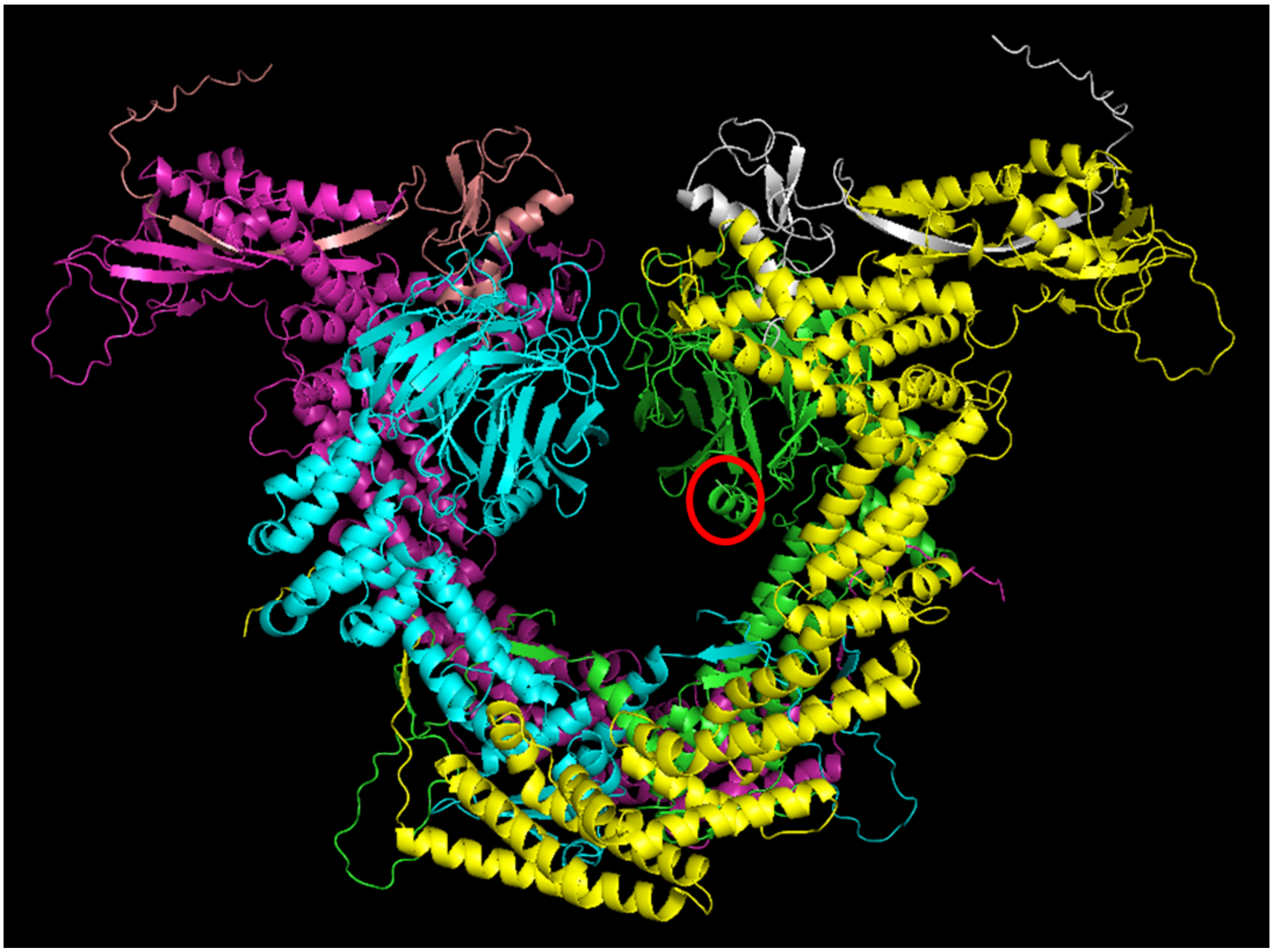

Figure S15. PDB structural representations of the KEAP1–CUL3–RBX1 dimer complex structure bind to the C-terminus of KEAP1.

16. Instructions for running Br-Matcher for Windows OS

1) Install Python (version 3.12.3).
2) Download the source code of Br-Matcher from the GitHub repository: https://github.com/ChenfengZhao/BrDTB.

data	3/20/2026 6:59 PM	File folder	
.gitignore	3/20/2026 6:59 PM	txtfile	1 KB
brdtb_cal	3/20/2026 6:59 PM	PY File	27 KB
LICENSE	3/20/2026 6:59 PM	File	12 KB
Readme.md	3/20/2026 6:59 PM	MD File	3 KB
Readme_win.md	3/20/2026 6:59 PM	MD File	2 KB
requirements	3/20/2026 6:59 PM	Text Document	1 KB

3) Copy the Excel files generated by Byonic Search and the mzXML mass spectrometry files converted by RawConverter into the data directory. An example of the contents of the data folder is shown in the figure below.

20250811_BBP_BrDTB_HEK293_1.mzXML	8/13/2025 11:22 AM	MZXML File	312,877 KB
20250811_BBP_BrDTB_HEK293_1.raw_202...	3/23/2026 4:02 PM	Microsoft Excel W...	1,133 KB

4) For the initial use, first create a virtual environment and install all required dependencies from requirements.txt. After activating the virtual environment, run the program from the Windows Command Prompt using the following command:

    a. *python brdtb_cal.py --data_excel 20250811_BBP_BrDTB_HEK293_1.raw_20250812_Byonic.xlsx*

    b. In this command, the --data_excel option specifies the Excel file to be processed.

5) Once the program finishes, the output files are generated in the ./results/ directory. Additional details are provided in the README file of the GitHub repository.

6) To run a specific peptide, open brdtb_cal.py. The dictionary variable pep_mod_dict (line 388) contains all defined modifications. You can add new modifications in this section.

```
387        # pep_mod_dict = {"+658.259" : "C30H43BrN8O4", "+57.021": "C2H3NO", "+15.995": "O"} # peptide modification dict {modification mass: modification composition}
388        pep_mod_dict = {"658.259" : "C30H43BrN8O4", "57.021": "C2H3NO", "15.995": "O", "485.172" : "C22H28BrN7O3S-1", "876.317" : "C42H53BrN8O8"}
389        abd_threshold = 0.005 # filter out the theoritical peaks that are lower than abd_threshold, reletive to the first theoritical peaks
390        rst_path = "./results"
391        rst_summary_fn = "result_summary.xlsx"
```

7) If the modification contains bromine (Br), add an elif branch at line 431. Otherwise, the peptide will be skipped during processing.

```
423         # # Ignore all the pipetides that don't contain [+658.259]
424         if "[+658.259]" in pep_n:
425             # print("INFO: [+658.259] found!")
426             pass
427         elif "[+485.172]" in pep_n:
428             # print("INFO: [+485.172] found!")
429             pass
430         elif "[+876.317]" in pep_n:
431             pass
432         else:
433             print("WARNING: [+658.259] or [+485.172] or [+876.317] not found! Skip the pipetide:", pep_n)
434             continue
```

Note: The contents of the website folder are identical to the GitHub repository.

17. RAW file information

Raw File Name	Cell line	Experiments	Related to figures
HEK293_Solu_5mM.raw	HEK293T	cysteines labeled with Br-DTB	Figure 2
HEK293_Solu_0.5mM.raw	HEK293T	cysteines labeled with Br-DTB	Figure 2
HEK293_Memb_5mM.raw	HEK293T	cysteines labeled with Br-DTB	Figure 2
HEK293_Memb_5mM.raw	HEK293T	cysteines labeled with Br-DTB	Figure 2
HeLa_Solu_5mM.raw	HeLa	cysteines labeled with Br-DTB	Figure 2
HeLa_Solu_0.5mM.raw	HeLa	cysteines labeled with Br-DTB	Figure 2
HeLa_Memb_5mM.raw	HeLa	cysteines labeled with Br-DTB	Figure 2
HeLa_Memb_5mM.raw	HeLa	cysteines labeled with Br-DTB	Figure 2
MCF7_Solu_5mM.raw	MCF-7	cysteines labeled with Br-DTB	Figure 2
MCF7_Solu_0.5mM.raw	MCF-7	cysteines labeled with Br-DTB	Figure 2
MCF7_Memb_5mM.raw	MCF-7	cysteines labeled with Br-DTB	Figure 2 (A)
MCF7_Memb_0.5mM.raw	MCF-7	cysteines labeled with Br-DTB	Figure 2
HepG2_Solu_5mM.raw	HepG2	cysteines labeled with Br-DTB	Figure 2
HepG2_Solu_0.5mM.raw	HepG2	cysteines labeled with Br-DTB	Figure 2

HepG2_Memb_5mM.raw	HepG2	cysteines labeled with Br-DTB	Figure 2
HepG2_Memb_5mM.raw	HepG2	cysteines labeled with Br-DTB	Figure 2
Jurkat_Solu_5mM.raw	Jurkat	cysteines labeled with Br-DTB	Figure 2
Jurkat_Solu_0.5mM.raw	Jurkat	cysteines labeled with Br-DTB	Figure 2
Jurkat_Memb_5mM.raw	Jurkat	cysteines labeled with Br-DTB	Figure 2
Jurkat_Memb_5mM.raw	Jurkat	cysteines labeled with Br-DTB	Figure 2
Synthetic_Peptide.raw	N/A	Synthetic Peptide MS	Figure S10
Synthetic Peptide_IAA.raw	N/A	Synthetic Peptide cysteine labeled AAI MS	Figure S10
Synthetic Peptide_IAA_BrDTB.raw	N/A	Synthetic Peptide cysteines labeled with Br-DTB	Figure S10
AHA_Solu_5mM.raw	HeLa	L-AHA labeled with Br-DTB	Figure 4
AHA_Insolu_5mM.raw	HeLa	L-AHA labeled with Br-DTB	Figure 4
Keap1.raw	HEK293T	PL-modified KEAP1 with BrDTB	Figure 4
Keap1_replicate.raw	HEK293T	PL-modified KEAP1 with BrDTB	Figure 4

18. Raw gels/blots

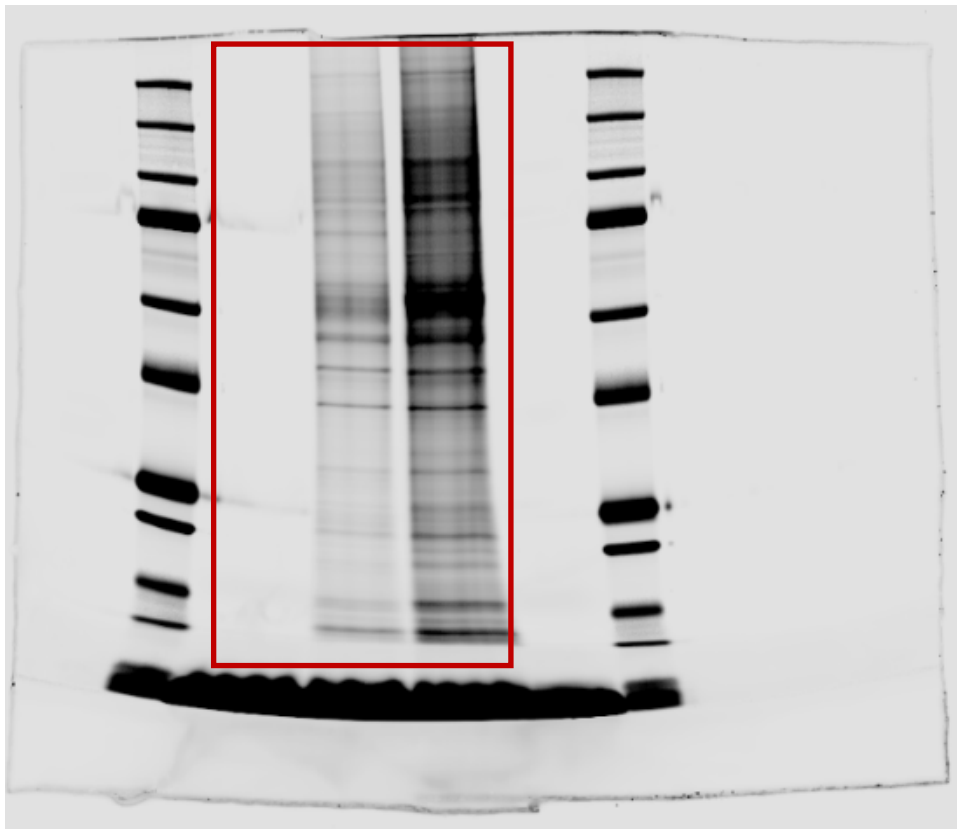

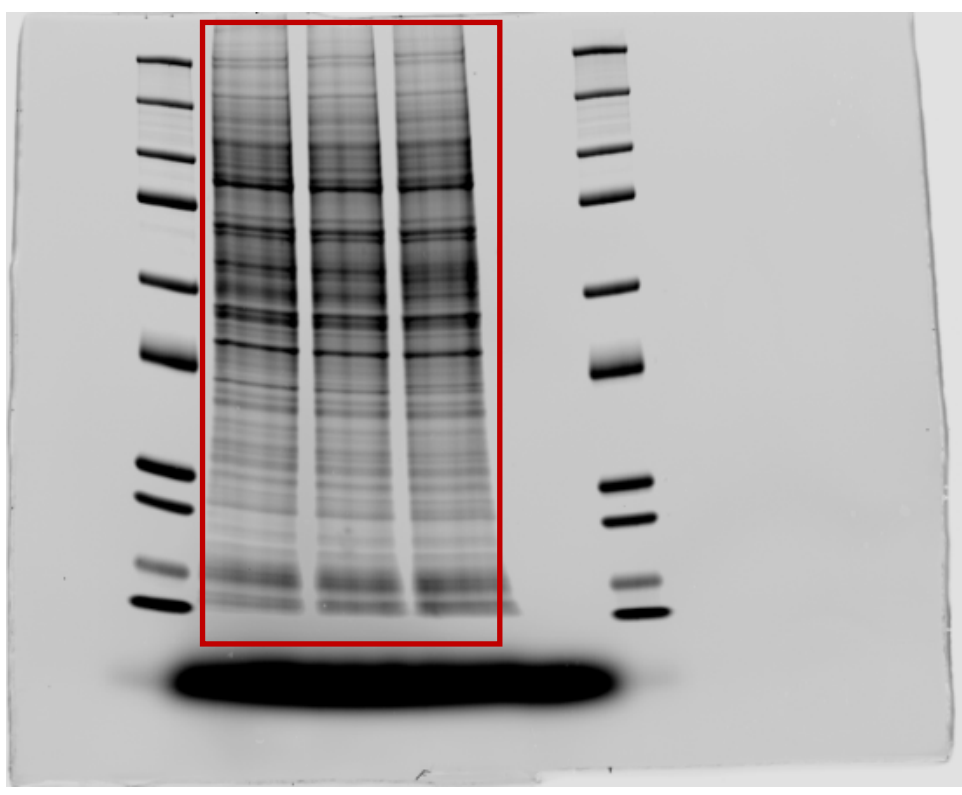

Raw images for Figure 1b.

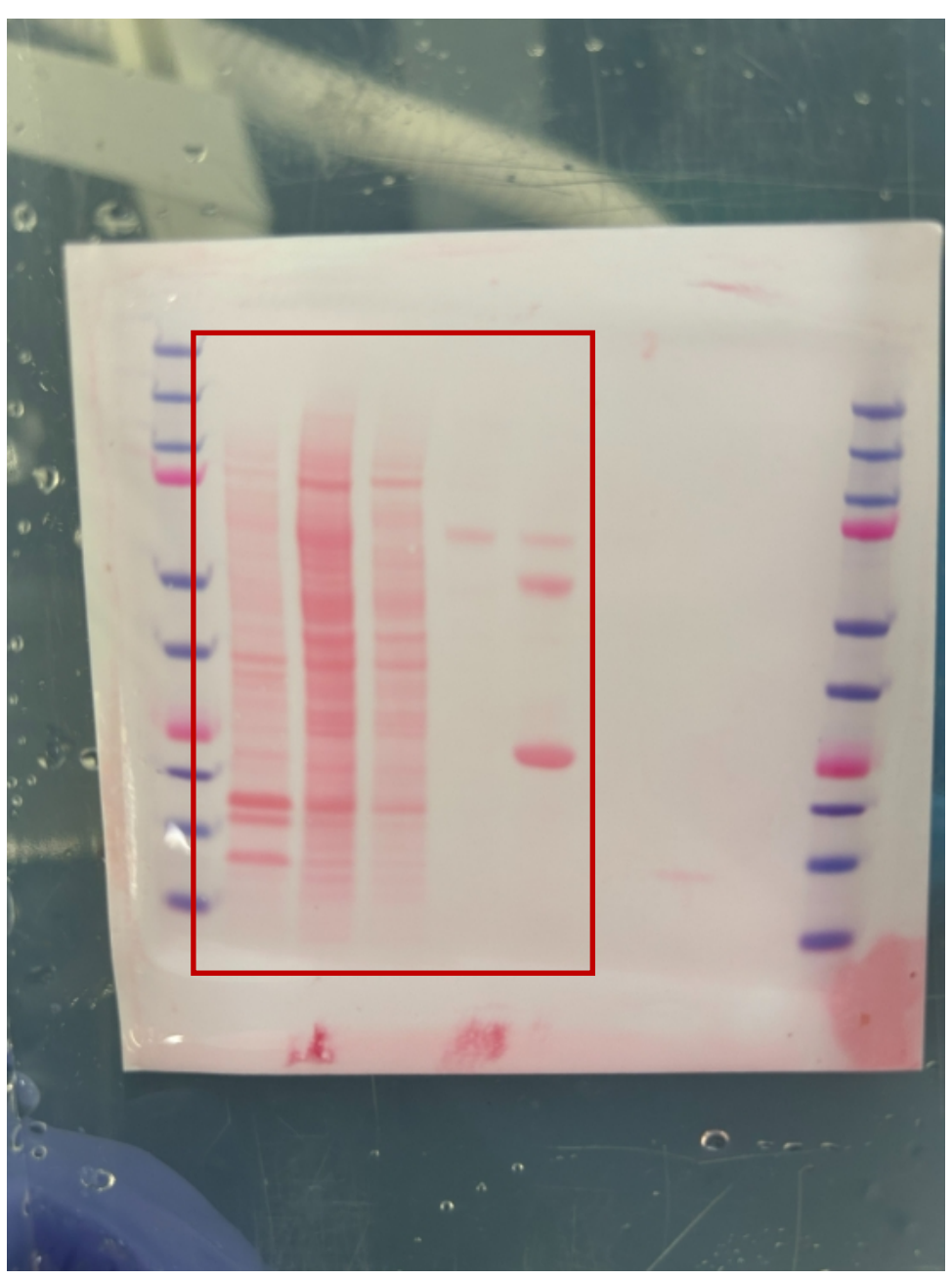

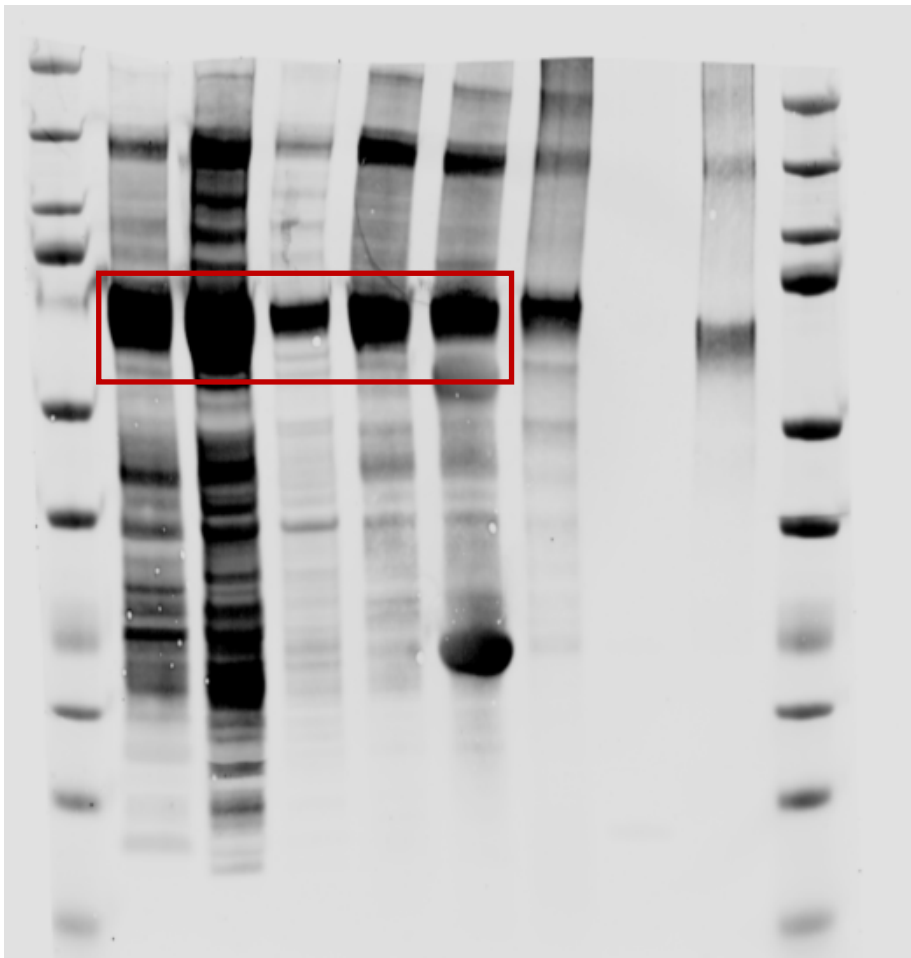

Raw images for Figure 4b.

19. **Click reaction efficiency check and Pulse-and-Chase Experiment: Br-DTB Labeling Followed by Rhodamine-Azide Click Reaction**

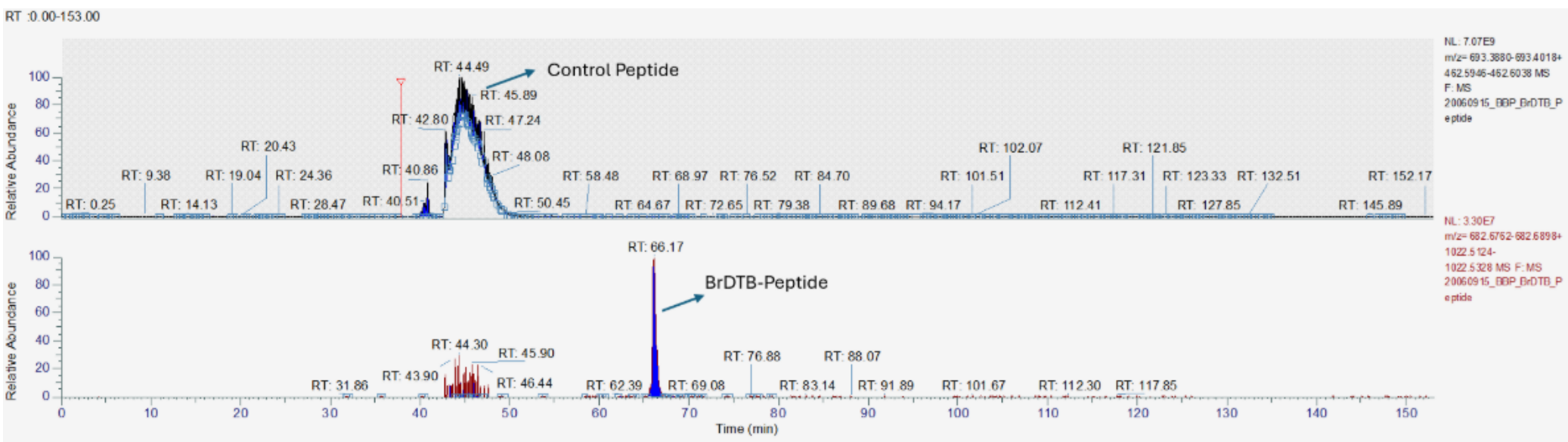

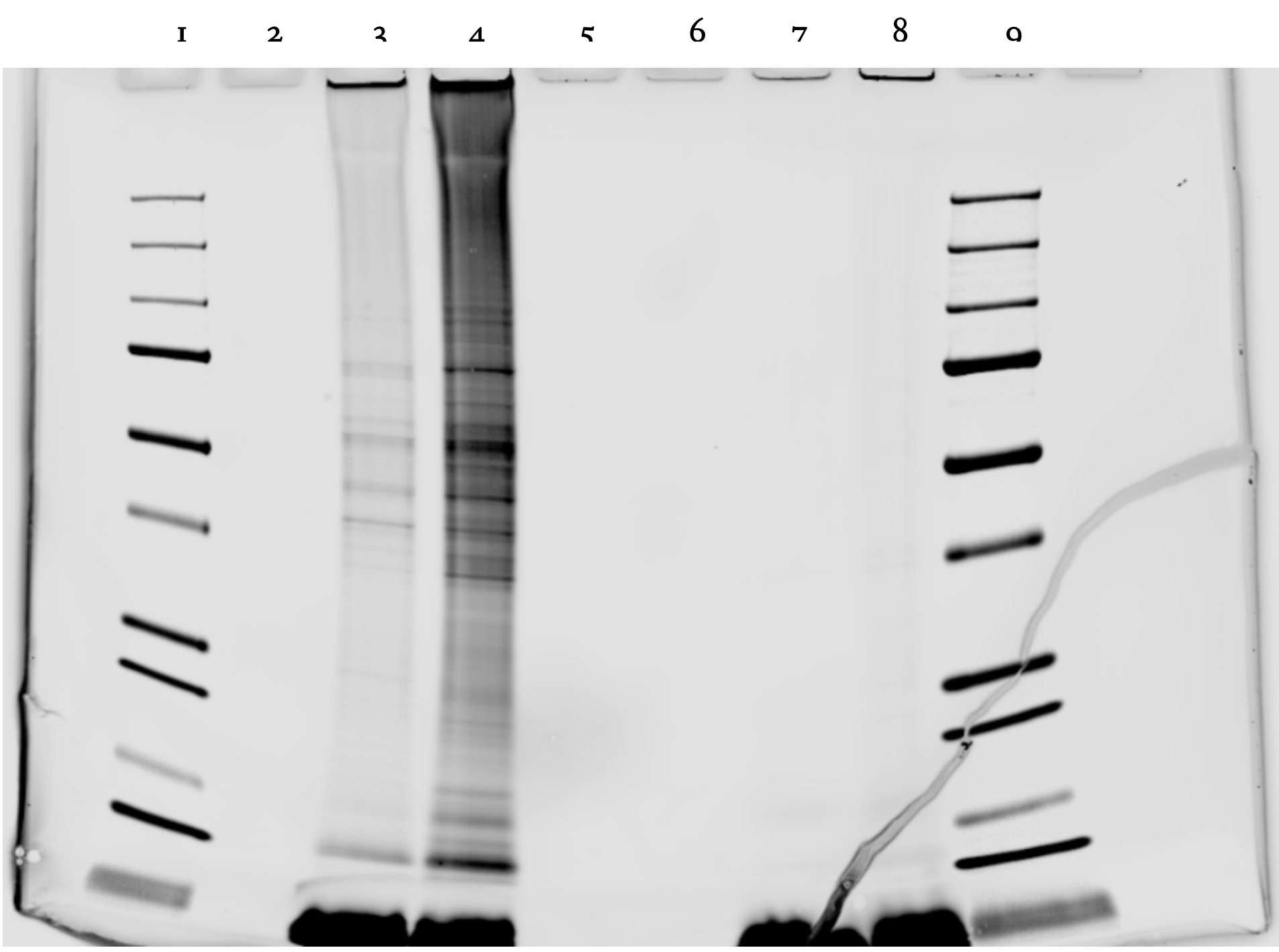

1. Ladder
2. DMSO
3. IAA 10uM+RhN3(click)
4. IAA 100uM+RhN3(click)

5. IAA 10 uM+BrDTB(Click)
6. IAA 100 uM+BrDTB(Click)
7. IAA 10 μM+BrDTB(Click-1)+ RhN3(Click-2)
8. IAA 100 μM+BrDTB(Click-1)+ RhN3(Click-2)
9. Ladder

Figure S16. Pulse-and-Chase Experiment: Br-DTB Labeling Followed by Rhodamine-Azide Click Reaction, fluorescence imaging.

Biological Replicates of Br-DTB Chemoproteomic Profiling

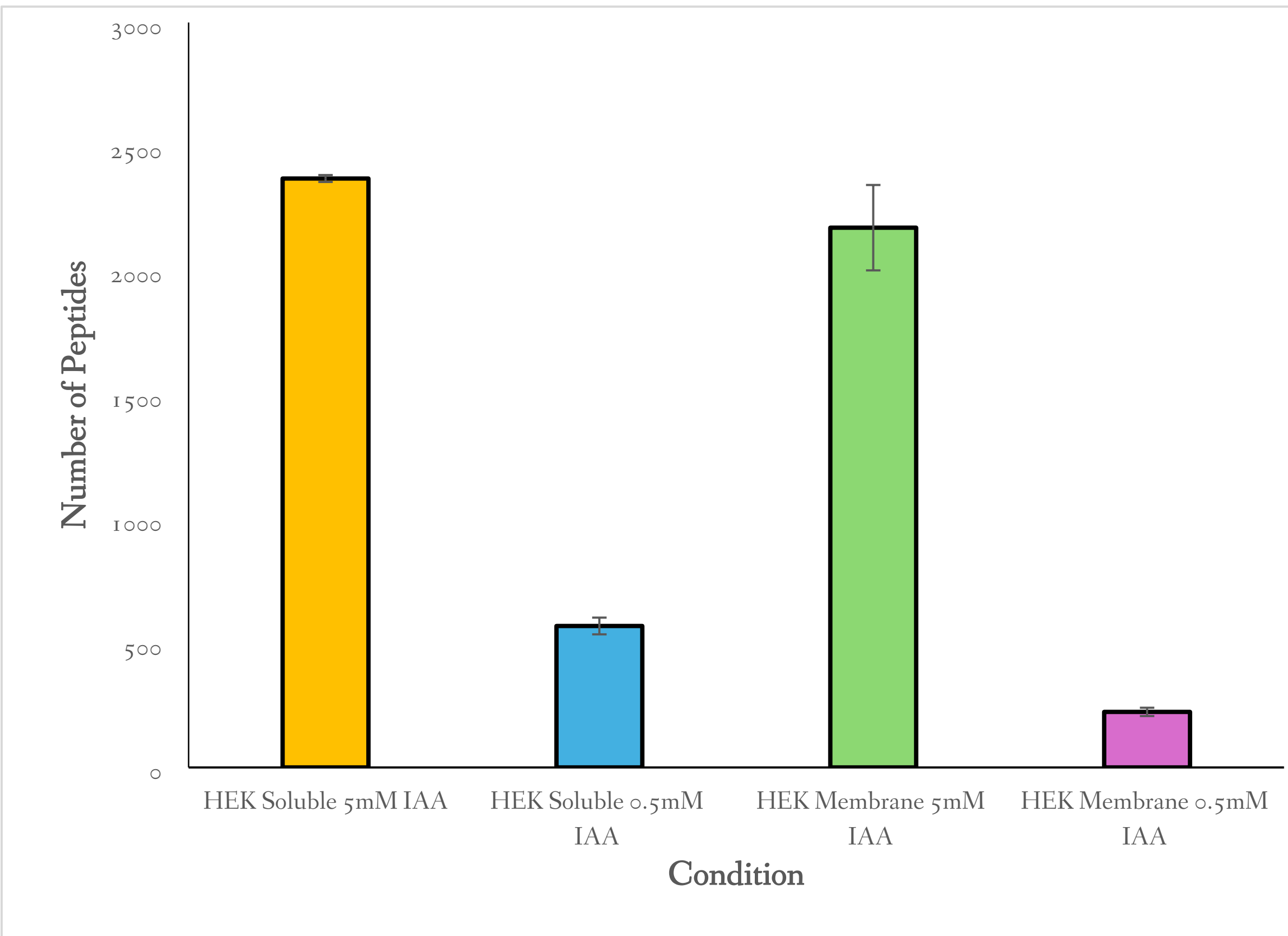

	Rep1	Rep2	Rep3	Average	St dev
HEK Soluble 5mM IAA	2388	2354	2373	2371.667	13.91242
HEK Soluble 0.5mM IAA	594	593	522	569.6667	33.7079
HEK Membrane 5mM IAA	2309	2281	1931	2173.667	171.9716
HEK Membrane 0.5mM IAA	229	241	201	223.6667	16.75974

Figure S17. Reproducibility of Peptide Identification in Br-DTB-Based Chemoproteomic Profiling of HEK293 Cells
